# Tumor microenvironment-triggered aggregation of semiconducting polymer nanochangers for self-programable theranostics of orthotopic glioma

**DOI:** 10.7150/thno.123074

**Published:** 2026-01-01

**Authors:** Zheming Song, Li Li, Anni Zhu, Danling Cheng, Yuyun Zhong, Zhiming Zheng, Cong Song, Jingchao Li, Zhong Guo

**Affiliations:** 1 State Key Laboratory of Advanced Fiber Materials, College of Biological Science and Medical Engineering, Donghua University, Shanghai 201620, China; 2 The Second Affiliated Hospital of Kunming Medical University, Key Laboratory of Neurological and Psychiatric Disease Research of Yunnan Province, Kunming 650223, China; 3 Shandong Provincial Hospital, Medical Science and Technology Innovation Center, Shandong First Medical University & Shandong Academy of Medical Sciences, Jinan 250117, China; 4 Center for Biological Science and Technology, Faculty of Arts and Sciences, Beijing Normal University, Zhuhai 519087, China

**Keywords:** Semiconducting polymer, aggregation, orthotopic glioma, nanotheranostics, tumor microenvironment

## Abstract

**Rationale:** Nanotheranostics have attracted significant research attention for their potential in improving glioma management through integrated diagnostic and therapeutic functions. However, the limited capacity for dynamic structural transformation of current nanotheranostics in the tumor microenvironment (TME) restricts theranostic outcomes.

**Methods:** Herein, we report a semiconducting polymer (SP)-based nanochanger (TM-P@SPN) that demonstrates aggregation-enhanced self-programable theranostics in orthotopic glioma upon glutathione (GSH) response. The TM-P@SPN is prepared using a SP as a triple-functional component (fluorescence probe, second near-infrared photoacoustic probe, and photothermal sensitizer), *β*-amyloid peptide domain (KLVFF)-linked PEG as an aggregation trigger switch, and transferrin modified manganese dioxide (TM) as a targeting theranostic agent.

**Results:** Upon GSH response in the TME, the TM-P@SPN disassembles to release PEG and Mn (II), enabling SP-KLVFF-mediated hydrophobic aggregation through hydrogen bonding, which consequently enhances both photoacoustic imaging (PAI) and photothermal therapy (PTT). Meanwhile, the released Mn(II) can be utilized for *T*_1_-weighted magnetic resonance imaging (MRI) and chemodynamic therapy (CDT). Moreover, both CDT- and PTT-induced immunogenic cell death effect and Mn(II)-activated STING pathway promote dendritic cells maturation, thereby triggering systemic immune effects.

**Conclusions:** This TME-responsive nanochanger is successfully used for self-programable theranostics, including fluorescence imaging (FLI)-enhanced PAI-MRI and CDT-enhanced PTT-immunotherapy.

## Introduction

Glioma is widely regarded as the most aggressive type of malignant tumor in the central nervous system. The current theranostics approaches for glioma are confronted with three major challenges. Firstly, the invasive growth pattern of glioma cells poses a major obstacle to complete surgical resection 1-5. Secondly, drug delivery efficacy is substantially limited by the blood-brain barrier (BBB) 6-9. Lastly and most critically, the glioma tumor microenvironment (TME), which is characterized by hypoxia, acidic pH, elevated reactive oxygen species (ROS), and altered glutathione (GSH) metabolism, along with immunosuppressive cell infiltration, collectively drives the poor prognosis and high recurrence rate of glioma following standard clinical treatment 10-12. Consequently, breakthrough theranostic strategies capable of concurrently addressing these challenges are urgently needed to achieve curative aims in glioma.

Recently, nanotheranostics have emerged as a novel and promising strategy for the diagnostics and therapeutics of glioma 13-17. Engineered with cell membrane coatings, peptide modifications and enhanced surface properties, these nanomaterials enable both BBB penetration and tumor-targeted accumulation while delivering therapeutic payloads and imaging agents with enhanced precision 18-20. Crucially, current nanotheranostics often respond intelligently to the TME, leading to stable drug release kinetics and improved imaging fidelity 21-25. A representative study by Xiao *et al*. 26 demonstrated that manganese dioxide (MnO_2_)/cisplatin-loaded nanogels achieved 94.0% Mn(II) release under TME-mimicking conditions, supporting chemotherapy and chemodynamic therapy (CDT) under magnetic resonance imaging (MRI) guidance for orthotopic glioma treatment. Similarly, Li *et al*. 20 developed a nanoprobe that undergoes swelling in the acidic TME, thereby accelerating ultrasound-triggered release of encapsulated therapeutics. This sequential process elicits potent immune response, achieving synergistic immuno-sonodynamic therapy against glioma.

Despite the significant advantages of TME-responsive nanotheranostics, the theranostic efficacy of current systems still requires substantial improvement. A key limitation lies in the predominantly unidirectional and irreversible structural transformations (e.g., carrier disintegration) of existing nanotheranostics, which cannot adapt to the dynamic evolution of TME, thereby constraining long-term treatment outcomes 27-29. This is particularly problematic in gliomas, where the BBB also significantly compromises nanotheranostics accumulation in tumor regions 30. Without relying on TME-triggered dynamic structural transformations of nanomaterials, further therapeutic enhancement would be unachievable. Therefore, next-generation nanotheranostics (e.g., shape-shifting nanoparticles or bioinspired molecular switches) should dynamically reconfigure their nanostructures in response to TME for precision theranostics.

Among numerous nanotheranostics, semiconducting polymers (SPs), with their remarkable optical properties, outstanding biocompatibility, and tunable chemical structures, have demonstrated extensive application potential in biomedical fields, particularly in photoacoustic imaging (PAI) and photothermal therapy (PTT) 31-34. Traditional SPs are limited to the near-infrared region I (NIR-I) window (700-900 nm), while NIR-II-optimized SPs (1000-1700 nm) exhibit superior tissue penetration and reduced scattering due to their longer excitation wavelengths 35-38. It has been well-established that enhancing SPs accumulation at tumor sites ensures both high-brightness imaging and efficient photothermal conversion 39-42. Recent advances focus on integrating SPs with other therapeutic and diagnostic modules, such as chemokinetic agents or immunomodulatory components, to construct multifunctional nanocomposites for enhanced diagnostic and therapeutic efficiency 43, 44. In our previous study, we developed a SP-based multifunctional nanoadjuvant that enabled tumor-specific on-demand release of the Toll-like receptor (TLR) agonist R848. This was achieved through NIR-II photothermal-triggered disruption of thermosensitive nanostructures under NIR-II irradiation, thereby synergizing PTT with TLR-mediated immunotherapy 45. However, the limited structural transformation capability of SP-based nanoparticles prevents amplification of signals within the TME niche, thereby hindering theranostics efficacy.

**Figure 1:**
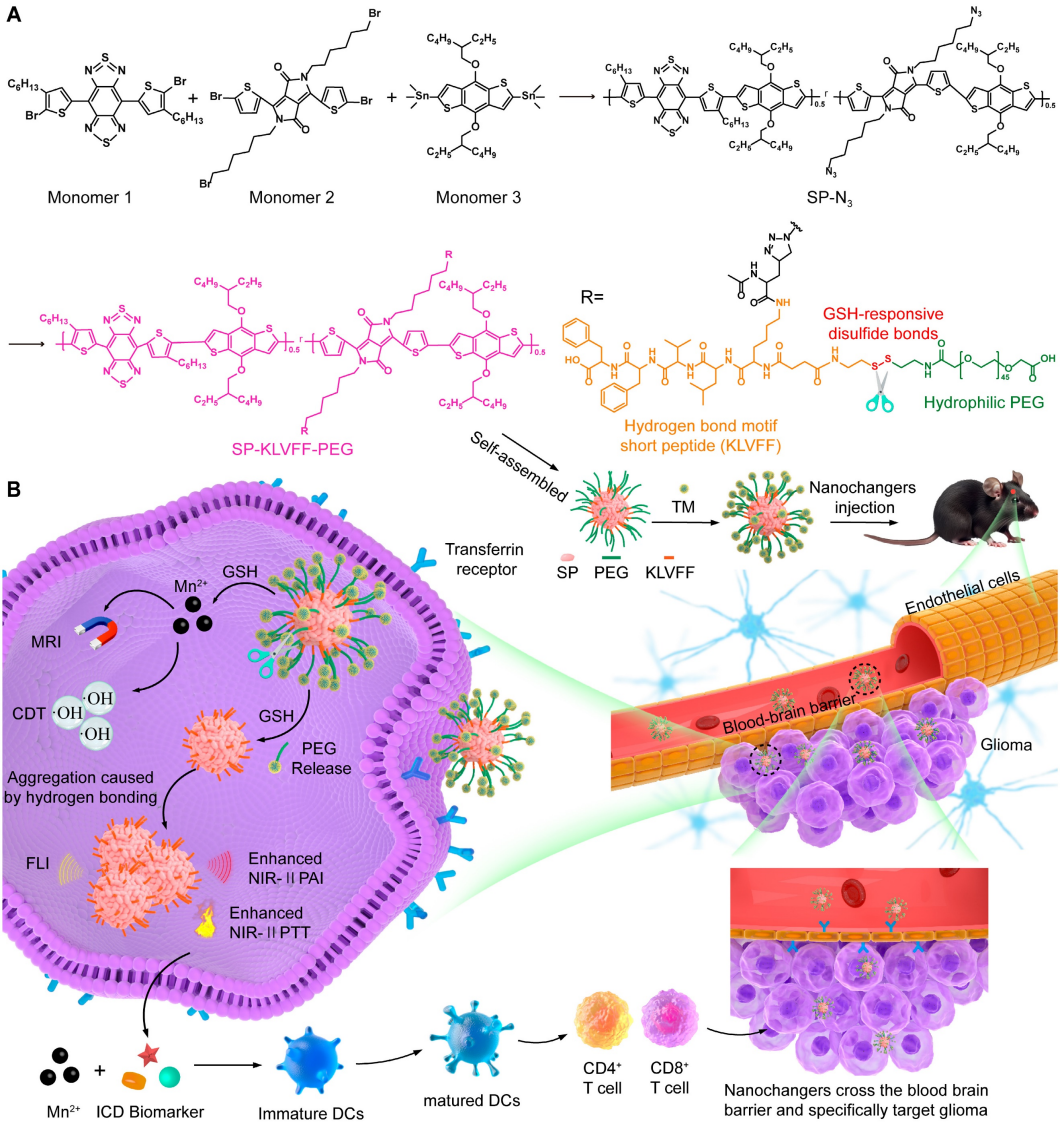
Diagram of TM-P@SPN for self-programable theranostics of orthotopic glioma. (A) Synthesis of TM-P@SPN nanochangers with tunable theranostic functionality. (B) Mechanism diagrams of the engineered TM-P@SPN nanochangers enabling tri-modal image-guided synergistic therapy for orthotopic glioma.

To further enhance theranostics efficacy, we propose an intelligent SP-based nanochanger for self-programable theranostics of orthotopic glioma through TME-responsive aggregation. As illustrated in Figure 1A, SP-based self-assemblies (P@SPN) were initially synthesized with a SP core as a triple-functional component (fluorescence probe, second near-infrared photoacoustic probe, and photothermal sensitizer) and a β-amyloid peptide domain (KLVFF) linked PEG *via* GSH responsive cystamine serving as the aggregation trigger switch. Whereafter, the transferrin modified manganese dioxide (TM) was prepared to decorate P@SPN, resulting in the synthesis of TM-P@SPN. Following transferrin-mediated BBB crossing and glioma targeting (Figure 1B), TM-P@SPN underwent GSH-triggered disassembly, releasing both PEG and Mn(II). Subsequently, the hydrophobic SP-KLVFF turned into aggregates *via* hydrogen bonding. In contrast to KLVFF-depleted control group (TM@SPN), the TM-P@SPN enhanced both PAI and PTT due to KLVFF-induced aggregation. Meanwhile, the released Mn(II) was utilized for *T*_1_-weighted MRI and CDT. Importantly, the combined CDT-PTT action induces potent immunogenic cell death (ICD) effect, while Mn(II) simultaneously activates the STING pathway to promote dendritic cell maturation and subsequent systemic immune activation. Through this innovative TME-triggered structural aggregation approach, TM-P@SPN enables comprehensive self-programable theranostics, integrating tri-modal imaging (fluorescence imaging (FLI), PAI and MRI) with triple-therapy (PTT, CDT, and immunotherapy) capabilities. To our knowledge, this work represents a novel strategy for TME-specific aggregation based on SPs, heralding the onset of a new era with substantially improved efficacy of nanotheranostics.

## Experimental Section

### Synthesis of SP-N_3_

A mixture of monomer 1 (10 mg), monomer 2 (9 mg), and monomer 3 (20 mg) was solubilized in 6 mL tetrahydrofuran (THF). Following deoxygenation, the solution underwent vigorous stirring at 100 °C (oil bath) for 1 h to afford the product SPN-Br. Subsequently, a mixture of SPN-Br (30 mg) and sodium azide (15 mg) was prepared in 10 mL of THF/DMF (2:1 v/v). After 12 h of stirring in the dark, SPN-N₃ was then extracted with saturated NaCl solution.

### Synthesis of KLVFF-s-s-PEG-COOH

The EDC coupling method was sufficiently employed for the preparation of KLVFF-PEG. Firstly, KLVFF-s-s-NH_2_ were formed. In brief, the DMSO solution containing EDC and NHS were dropwise added to KLVFF peptides dissolved in DMSO under stirring for activating carboxyl groups. The resulting mixture was infused dropwise into a DMSO solution containing cystamine and stirred vigorously at ambient temperature for 72 h. Among them, the molar ratio of KLVFF, NHS, EDC, and cystamine was maintained at 1:10:10:10 during feeding. The reaction product was dialyzed against water (6 times, 2 L) and freeze-dried into KLVFF-s-s-NH_2_ powder. Subsequently, KLVFF-PEG were synthesized through similar steps, except that the reagents used were KLVFF-s-s-NH_2_, PEG-COOH, EDC, and NHS, respectively. Among them, the molar ratio of them were 1:10:50:50. And after dialysis, the product was centrifuged (1000 rpm/min, 10 min) to eliminate precipitates and then freeze-dried into powder.

### Synthesis of P@SPN and SPN

The P@SPN was assembled based on SP-N_3_. Concretely speaking, SP-N_3_ (0.5 mg), KLVFF-PEG (2 mg), CuBr (2.2 mg), and PMDETA (13.4 mg) were mixed and stirred in the solution of THF/DMSO (5 mL in total, v/v = 1: 1) under nitrogen atmosphere for 3 days. The crude product was dialyzed against water (6 times, 2 L) and freeze-dried into P@SPN powder. In the same method, The SPN was assembled by alkynyl-PEG-COOH (3 mg) instead of KLVFF-PEG.

### Preparation of TM-P@SPN and TM@SPN

Frist, transferrin (Tf, 62.5 mg), and KMnO₄ (7.9 mg) were mixed and stirred in aqueous phase. After 6 h, The reaction product was dialyzed against water (6 times, 2 L) to obtain TM solution. Second, P@SPN (2 mg), EDC (11.4 mg) and NHS (7.0 mg) were mixed and stirred in aqueous phase for 30 min to activate carboxyl groups. Third, the obtained TM solution was dropwise added into the activate P@SPN under vigorous stirring. After 12 h, the crude product was finally dialyzed against water (6 times, 2 L) to gain TM-P@SPN. Similarly, the TM@SPN was obtained by using SPN (2 mg) instead of P@SPN.

### OH generation via fenton-Like reaction

Solutions of H_2_O_2_ (100 μM), GSH (10 mM), methylene blue (MB, 10 μg/mL), TM@SPN and TM-P@SPN ([Mn] = 10 μg/mL) were first prepared, respectively. Equal volumes of the solutions were sequentially mixed according to the following groups: TM-P@SPN + GSH + H_2_O_2_ + MB, TM@SPN + GSH + H_2_O_2_ + MB, TM-P@SPN + H_2_O_2_ + MB, TM@SPN + H_2_O_2_ + MB, H_2_O_2_ + MB, MB and H_2_O_2_. UV-vis spectral measurements were taken after 30 min to assess MB concentration changes through absorbance at 650 nm.

### Evaluation of photothermal performance

Samples of TM@SPN and TM-P@SPN ([SP] = 100 μg/mL) in PBS solutions were irradiated for 5 min using a 1064 nm NIR-II laser system operating at 1.0 W/cm^2^, and then cooled for 5 minutes. The above steps were repeated five times, and the temperature changes were recorded. To quantify the photothermal conversion efficiency (PCE) of TM-P@SPN and TM@SPN, heating and cooling cycles of the aqueous dispersion were performed under NIR laser irradiation, and the PCE value was calculated according to established literature protocols 46. For comparison, the widely used clinical agent indocyanine green (ICG) was measured under identical conditions. Subsequently, Samples of TM@SPN and TM-P@SPN ([SP] = 100 μg/mL) in PBS solutions were irradiated for 5 min using a 1064 nm NIR-II laser system operating at 0.6, 0.9, and 1.2 W/cm^2^, separately, and the temperature changes were recorded over 10 minutes. In addition, TM@SPN and TM-P@SPN in PBS solutions at SP concentrations ranging from 25 to 100 μg/mL were irradiated for 5 min using a 1064 nm NIR-II laser system operating at 1.0 W/cm^2^, with temperature variations monitored for a 10-minute duration. Notably, 10 mM GSH had been added into each solution before each irradiation.

### *In vitro* evaluations

The murine glioma cell line (GL261 cells), murine dendritic cells (DCs), and murine brain microvascular endothelial cell line (bEnd.3 cells) were passaged as needed for *in vitro* experiments including cytotoxicity, blood-brain barrier penetration, cellular uptake, photothermal therapeutic effect, GSH depletion, ROS generation, ICD effect, and interferon-*β* (IFN-*β*) level.

### Animal experiments

All animal procedures were conducted in compliance with the ethical guidelines approved by the Animal Care and Use Committee of Donghua University (Approve number: DHUEC-NSFC-2022-16). An orthotopic glioma model of mice was built for biodistribution and imaging and therapy evaluations* in vivo*. The biosafety of TM-P@SPN were assessed by histological examinations of major organs.

### Statistical analysis

All experimental data are expressed as mean ± standard deviation from a minimum of three independent replicates. Statistical significance was determined by one-way ANOVA using IBM SPSS Statistics 25 software (IBM, Armonk, NY), with the following thresholds: * for *p* < 0.05, ** for *p* < 0.01, and *** for *p* < 0.001, respectively.

## Results and Discussion

### Synthesis and properties of TM-P@SPN and TM@SPN

In this work, we developed a GSH-responsive nanochanger (TM-P@SPN) based on SPs coupled with both KLVFF-PEG and TM. First of all, SP-N_3_ was synthesized according to a previously reported method, 47 with its successful formation verified by ^1^H nuclear magnetic resonance (NMR) spectroscopy (Figure S1), Gel Permeation Chromatography (GPC) (Figure S2) and Fourier-Transform Infrared (FT-IR) spectroscopy (Figure S3). Figure S2 reveals that GPC analysis confirms the successful synthesis of the SP-N_3_, showing a unimodal distribution characterized by a single peak accounting for 100% of the total peak area. This indicates that all molecular species fall within a continuous molecular weight range, with a peak molecular weight of 567 Da. The FT-IR spectrum clearly shows the characteristic azide (-N_3_) absorption peak at ~2100 cm^-1^, providing conclusive evidence for the successful introduction of the azide group. And then, KLVFF-PEG was synthesized and characterized by NMR spectroscopy. The characteristic peak related with NH could be detected in ^1^H NMR (Figure S4), revealing that its successful synthesis. Lastly, the content of manganese (Mn) in the final nanoparticle TM-P@SPN and its control product TM@SPN (without KLVFF linkage) was quantified. The Mn concentrations in 25 mg/L solutions of TM-P@SPN and TM@SPN were measured to be 3.05 mg/L and 2.55 mg/L, respectively (Figure S5). The difference in Mn^2+^ loading capacity between TM-P@SPN and TM@SPN may be attributed to their distinct surface architectures. The denser SP polymer arrangement on TM@SPN could impose greater steric hindrance, limiting TM conjugation efficiency. In contrast, the presence of the flexible KLVFF peptide in TM-P@SPN may extend further from the nanochanger surface, potentially reducing steric constraints and facilitating higher TM binding and Mn^2+^ incorporation. This structural distinction highlights the role of nanochanger design in modulating therapeutic agent loading.

**Figure 2:**
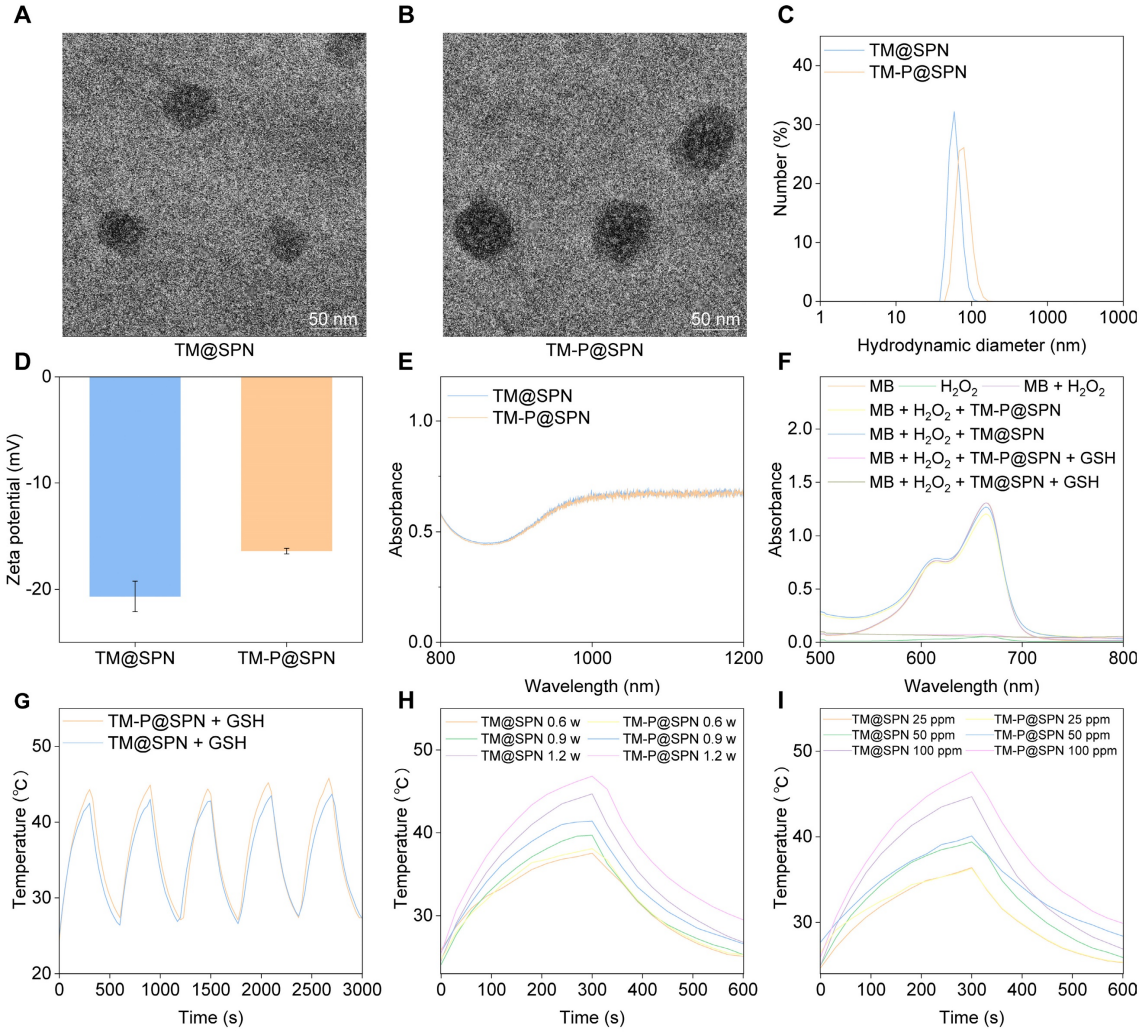
Characterization of SP-based nanochangers. TEM images of TM@SPN (A) and TM-P@SPN (B). Hydrodynamic sizes (C) and zeta potentials (D) of TM@SPN and TM-P@SPN (n = 3). Data presented as mean ± SD. (E) Comparative NIR absorption spectra (800-1200 nm) of TM@SPN and TM-P@SPN. (F) UV-vis spectra of MB solutions treated with different groups. (G) Temperature profiles of TM@SPN and TM-P@SPN during heating and cooling over 5 laser on/off cycles. (H) Temperature curves of TM@SPN and TM-P@SPN upon same SP concentration under NIR-II laser irradiation at graded power densities with GSH supplementation. (I) Temperature curves of TM@SPN and TM-P@SPN at different SP concentrations upon same laser irradiation. For H and I, the curves shown are representative of three independent experiments.

The nanochanger TM-P@SPN and its control TM@SPN were further characterized using multiple analytical techniques. As revealed by high-resolution transmission electron microscope (TEM) (Figure 2A-B), both TM@SPN and TM-P@SPN displayed monodisperse spherical morphologies and uniform distribution with dry-state diameters of 68.7 ± 3.5 nm (TM@SPN) and 82.3 ± 4.1 nm (TM-P@SPN). Dynamic Light Scattering measurements yielded hydrodynamic diameters of 72.0 ± 1.9 nm (PDI 0.30 ± 0.02) for TM@SPN and 98.5 ± 2.8 nm (PDI 0.32 ± 0.02) for TM-P@SPN. (Figure 2C and Figure S6). The greater hydrodynamic size of TM-P@SPN may be attributed to is the functionalization of *β*-amyloid peptide-KLVFF. To directly validate the central hypothesis of SP-KLVFF-mediated hydrophobic aggregation, we further investigated the GSH-responsive aggregation behavior. The hydrodynamic sizes of TM@SPN and TM-P@SPN in GSH-containing solutions were monitored by DLS. TM-P@SPN exhibited a concentration-dependent increase in diameter, reaching 250 - 300 nm at 2.5 μM GSH and 500 - 600 nm at 5 μM GSH, whereas TM@SPN remained nearly unchanged across all GSH concentrations (Figure S7). These results confirm the GSH-triggered aggregation behavior specific to TM-P@SPN. Corresponding TEM observations of TM-P@SPN further corroborated the formation of large aggregates under high GSH conditions, providing visual evidence of the morphological changes (Figure S8). These results characterize the GSH-responsive aggregation kinetics of TM-P@SPN and offer direct experimental support for the proposed mechanism of enhanced theranostic efficacy. Due to the modification of TM, the zeta potentials of both TM@SPN and TM-P@SPN were negative, which were -20.5 ± 0.6 mV and -17.6 ± 0.8 mV, respectively (Figure 2D). UV-Vis spectroscopy confirmed that both TM@SPN and TM-P@SPN retained strong NIR-II absorption profiles (1000-1200 nm) (Figure 2E). And fluorescence spectra exhibited that they possessed bright emission properties, supporting their fluorescence imaging performance (Figure S9).

The •OH generation abilities of TM@SPN or TM-P@SPN were checked using a MB degradation experiment. As displayed in Figure 2F, the decrease in absorbance at 664 nm for both TM@SPN and TM-P@SPN in the presence of both GSH and H_2_O_2_ suggested •OH generation. Considering the distinct characteristics of the TME,^12^ both TM@SPN and TM-P@SPN are considered to have the potential to undergo CDT *via* Mn(II)-mediated Fenton-like reactions. The hemolytic properties of TM@SPN and TM-P@SPN were also assessed using erythrocytes. The hemolysis rate of neither TM@SPN nor TM-P@SPN exceeded 5.0% at any tested concentration, proving their satisfactory hemocompatibility (Figure S10). Subsequently, the long-term colloidal stability of TM-P@SPN was systematically assessed. Over the 14-day monitoring period, the hydrodynamic diameters of TM-P@SPN remained within narrow ranges: 75.1 - 77.8 nm (PDI: 0.29 - 0.33) in H_2_O, 77.7 -78.2 nm (PDI: 0.24 - 0.26) in PBS, and 79.1 - 82.5 nm (PDI: 0.28 - 0.30) in PBS containing 10% fetal bovine serum (FBS), respectively (Figure S11). No significant aggregation or precipitation was observed throughout the study. Additionally, UV-Vis-NIR absorption profiles of TM-P@SPN in H_2_O were measured over a 14-day period (Figure S12). The nearly unchanged UV-Vis-NIR absorption profiles at Day 0, 7, and 14 further confirm the excellent stability of TM-P@SPN. This high colloidal stability confirms that the nanostructures remain intact during storage and under physiological conditions, which is crucial for ensuring effective targeted delivery to the tumor site.

Next, the temperature performance of TM@SPN and TM-P@SPN was evaluated by monitoring their temperature fluctuations during 1064 nm laser exposure. As demonstrated in Figure 2G, the temperature values of both TM@SPN and TM-P@SPN exceeded 42 °C within five minutes under laser irradiation (1.0 W/cm^2^). Identical temperature trends were observed over 5 laser on/off cycles, indicating stable photothermal performance. TM-P@SPN showed enhanced heating capability (ΔT = 21.3°C *vs*. 19.7°C for TM@SPN). Most importantly, the photothermal conversion efficiency (PCE) was quantitatively determined to be 45.6% for TM-P@SPN, significantly higher than that of TM@SPN (38.5%) and the common clinical agent ICG (22.5%) under the same measurement conditions (Figure S13). This enhancement is attributed to the GSH-responsive disassembly of TM-P@SPN, which releases PEG and triggers SP-KLVFF-mediated hydrophobic aggregation through hydrogen bonding, thereby enhancing photothermal conversion. The temperature fluctuations of both TM@SPN and TM-P@SPN were also recorded under various irradiation power and concentration. The peak temperatures of both TM@SPN and TM-P@SPN increase as the irradiation power rises when the SP concentration of both TM@SPN and TM-P@SPN were set at 100 μg/mL (Figure 2H). Similarly, the peak temperatures of both TM@SPN and TM-P@SPN increase with rising concentration at a fixed irradiation power (1.0 W/cm^2^) (Figure 2I). Importantly, in the presence of GSH, the resulting aggregation of SP-KLVFF ensures that TM-P@SPN consistently demonstrates superior photothermal performance compared to TM@SPN under equivalent conditions.

### Evaluation of nanochangers effects *in vitro*

**Figure 3:**
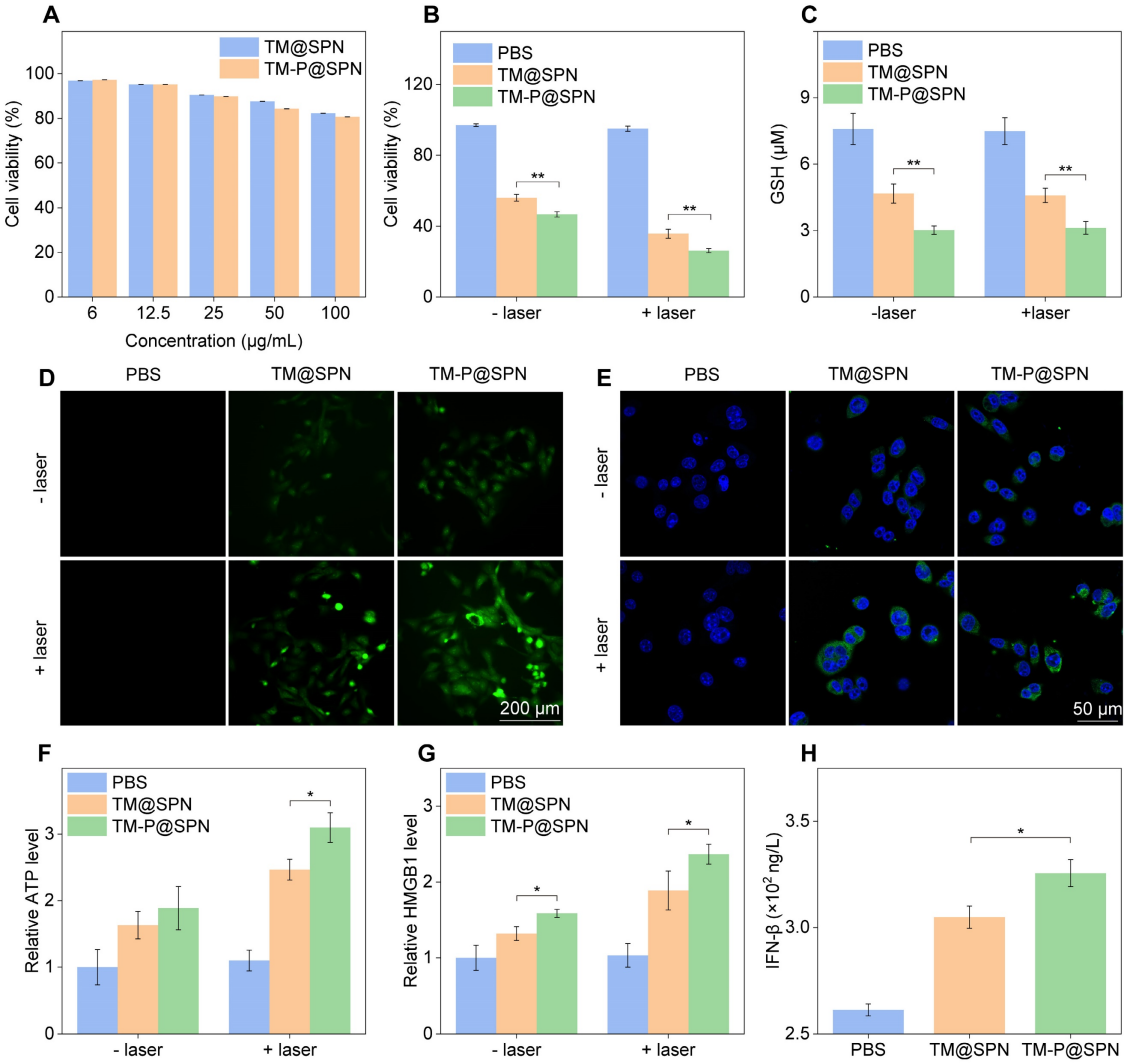
Evaluation of nanochangers effects *in vitro*. (A) Cytotoxicity assessment of GL261 cells treated with TM@SPN or TM-P@SPN at various SP concentrations (n = 3). Cell Viability (B), intracellular GSH content (C), ROS level (D), CRT expression (E), ATP secretion (F), and HMGB1 release (G) of GL261 cells treated with TM@SPN or TM-P@SPN in an H_2_O_2_-rich environment, with or without laser irradiation (n = 3). (H) IFN-*β* secretion of DCs treated with TM@SPN or TM-P@SPN in an H_2_O_2_-rich environment under laser irradiation (n = 3). Data presented as mean ± SD.

Prior to evaluating the* in vitro* therapeutic effects, the cytotoxicity, BBB-crossing capacity, and glioma-targeting performance of the nanochanger TM-P@SPN were first investigated. As shown in Figure 3A, GL261 cells sustained above 80.6% viability after 24 h treatment with TM-P@SPN or its control TM@SPN in a SP concentration range 0-100 μg/mL, confirming negligible cytotoxicity of both TM@SPN and TM-P@SPN. Subsequently, the BBB penetration capacity of TM-P@SPN was evaluated using an *in vitro* BBB model. Figure S14 illustrates the model composition and nanochanger delivery protocol, while Figure S15 presents the corresponding results. The images from confocal laser scanning microscopy (CLSM) revealed comparable fluorescence intensities between the nanochanger TM-P@SPN and the control TM@SPN in the underlying GL261 cells. After calculation, the penetration rate of TM-P@SPN and TM@SPN was 16.2% and 15.0%, respectively. The BBB penetration observed for both TM-P@SPN and TM@SPN benefits from the well-established role of Tf functionalization in promoting transport across brain endothelial barriers. This effect is widely attributed to transferrin receptor (TfR)-mediated transcytosis, a highly efficient and specific mechanism for targeted brain delivery that has been extensively reported in previous studies 48, 49.

Next, the glioma-targeting ability of TM@SPN and TM-P@SPN was evaluated. Flow cytometry analysis revealed nearly identical fluorescence intensities (Figure S16), indicating comparable cellular uptake by GL261 cells. This phenomenon can also be attributed to their similar degree of Tf modification. As reported in the literature, Tf-decorated nanoparticles can enter cells *via* TfR-mediated endocytosis 18. The specific mechanism involves the binding of Tf to TfR highly expressed on the GL261 cell membrane, which initiates clathrin-mediated endocytosis and leads to the formation of endosomes. Within the acidic environment of the endosome, Tf dissociates from TfR, facilitating the subsequent trafficking of nanoparticles to lysosomes, thereby achieving efficient cellular internalization 49. Adjacently, we evaluated the intracellular localization and trafficking of TM@SPN and TM-P@SPN by co-staining lysosomes with LysoTracker Green. As shown in Figure S17A-B, significant co-localization was observed for both TM@SPN and TM-P@SPN with lysosomes after 3, 6, 9, and 12 hours of incubation, suggesting effective internalization likely mediated by Tf-targeted uptake. Notably, a gradual decrease in Pearson's correlation coefficient (PCC) over time was observed, indicating lysosomal escape (Figure S17C). TM-P@SPN consistently exhibited lower PCC values compared to TM@SPN at all time points, suggesting more efficient escape. We hypothesize that under the high-acidity and high-GSH conditions within lysosomes, TM-P@SPN undergoes disassembly, releasing hydrophobic KLVFF peptides that trigger self-aggregation and physically disrupt the lysosomal membrane, thereby promoting enhanced escape. In summary, these data confirm that both TM@SPN and TM-P@SPN enter cells* via* TfR-mediated endocytosis and localize within lysosomes, while TM-P@SPN demonstrates superior lysosomal escape capability due to its responsive assembly property. Similarly, after co-incubating TM@SPN or TM-P@SPN with GL261 cells for 12 hours, co-localization analysis with mitochondrial markers revealed no significant overlap between the mitochondrial fluorescence signals and those of the TM@SPN or TM-P@SPN (Figure S18). This indicates that TM@SPN and TM-P@SPN likely exert their functions intracellularly through lysosomal escape pathways rather than *via* direct interaction with mitochondria 50, 51.

The combinatorial therapeutic efficacy of Mn(II)-based CDT and NIR laser-induced PTT was systematically examined in glioma cell models using TM-P@SPN. To rule out potential confounding effects of the H_2_O_2_-enriched TME-mimicking condition, we first confirmed that 100 μM H_2_O_2_ alone did not significantly affect cell viability (98.5% viability *vs.* 100% in 0 μM H_2_O_2_ control, Figure S19), ensuring that subsequent therapeutic effects were attributable to the nanochangers. Subsequently, the viability of cells after incubation with TM-P@SPN was evaluated by Cell Counting Kit-8 (CCK-8) assay under conditions mimicking the H_2_O_2_-enriched TME. As shown in Figure 3B, both TM-P@SPN and the control TM@SPN exhibited significant cell inhibition regardless of laser treatment. Without laser irradiation, the cell viability was 58.4% for TM@SPN and 50.1% for TM-P@SPN, primarily attributed to Mn(II)-mediated Fenton-like reactions. While laser-activated PTT significantly improved therapeutic outcomes, as evidenced by cell viability rates of 35.2% (TM@SPN + laser) compared to 26.2% (TM-P@SPN + laser) (*p* < 0.01). To further elucidate the mode of cell death, Annexin V-FITC/PI staining was performed and analyzed by flow cytometry (Figure S20). The PBS and PBS + laser groups showed minimal background apoptosis (less than 5%). Treatment with TM@SPN and TM-P@SPN alone induced apoptosis in 24.9% and 29.2% of cells, respectively, confirming the cytotoxic effects of CDT. Most notably, the combinatorial therapy dramatically enhanced cell killing, with the apoptotic rate increasing to 33.1% for TM@SPN + laser and 41.0% for TM-P@SPN + laser. Similarly, a live/dead cell staining assay (Calcein-AM/PI) was also conducted to corroborate these findings at the single-cell level. The quantitative flow cytometric analysis revealed a consistent trend with the apoptosis assay results (Figure S21): The TM-P@SPN + laser group exhibited the highest proportion of dead cells, followed by TM@SPN + laser, while control groups (PBS and PBS + laser) showed minimal cell death. The lower cell viability and higher cell mortality rate of TM-P@SPN plus laser irradiation directly corroborated the SP-KLVFF-mediated hydrophobic aggregation mechanism for amplified photothermal therapeutic effect.

Next, the oxidative stress in GL261 cells following TM-P@SPN treatment with laser irradiation was analyzed. As shown in Figure 3C, regardless of laser irradiation, the prepared TM-P@SPN revealed the phenomenon of GSH consumption owing to manganese-mediated Fenton-like reaction. Notably, GL261 cells treated with TM-P@SPN demonstrated a higher GSH consumption than that of TM@SPN, which was due to the higher manganese content in TM-P@SPN. Subsequently, the effect of the TM-P@SPN on the intracellular ROS levels was investigated by inverted fluorescence microscope. Compared with the PBS and TM@SPN groups, the TM-P@SPN group exhibited stronger cellular fluorescence intensity, which was further enhanced by laser irradiation (Figure 3D and Figure S22). SP-KLVFF-mediated hydrophobic aggregation likely enhanced photothermal conversion, improving PTT efficacy.

The ICD effect of the *in vitro* nanochanger treatments combined with laser irradiation was also examined. CLSM images displayed significantly higher calreticulin (CRT) expression level in GL261 cells incubated with TM-P@SPN than TM@SPN, with laser irradiation further enhancing CRT levels (*p* < 0.01) (Figure 3E and Figure S23). Similarly, ATP secretion and HMGB1 release levels in group TM-P@SPN were also higher than TM@SPN, especially post-laser treatment (*p* < 0.05) (Figure 3F-G). In brief, the ICD effect of TM-P@SPN was more pronounced than TM@SPN, owing to SP-KLVFF-mediated hydrophobic aggregation that enhances PTT efficacy following GSH-responsive disulfide cleavage. Additionally, both TM@SPN and TM-P@SPN could increase the secretion level of IFN-*β* in DCs upon laser irradiation (Figure 3H), suggesting that both TM@SPN and TM-P@SPN can mediate activation of STING pathway owing to the presence of Mn(II). Moreover, TM-P@SPN induced significantly higher IFN-β production than TM@SPN (*p* < 0.05), likely due to its greater manganese content at equal mass concentrations.

### Imaging evaluation of orthotopic glioma

**Figure 4:**
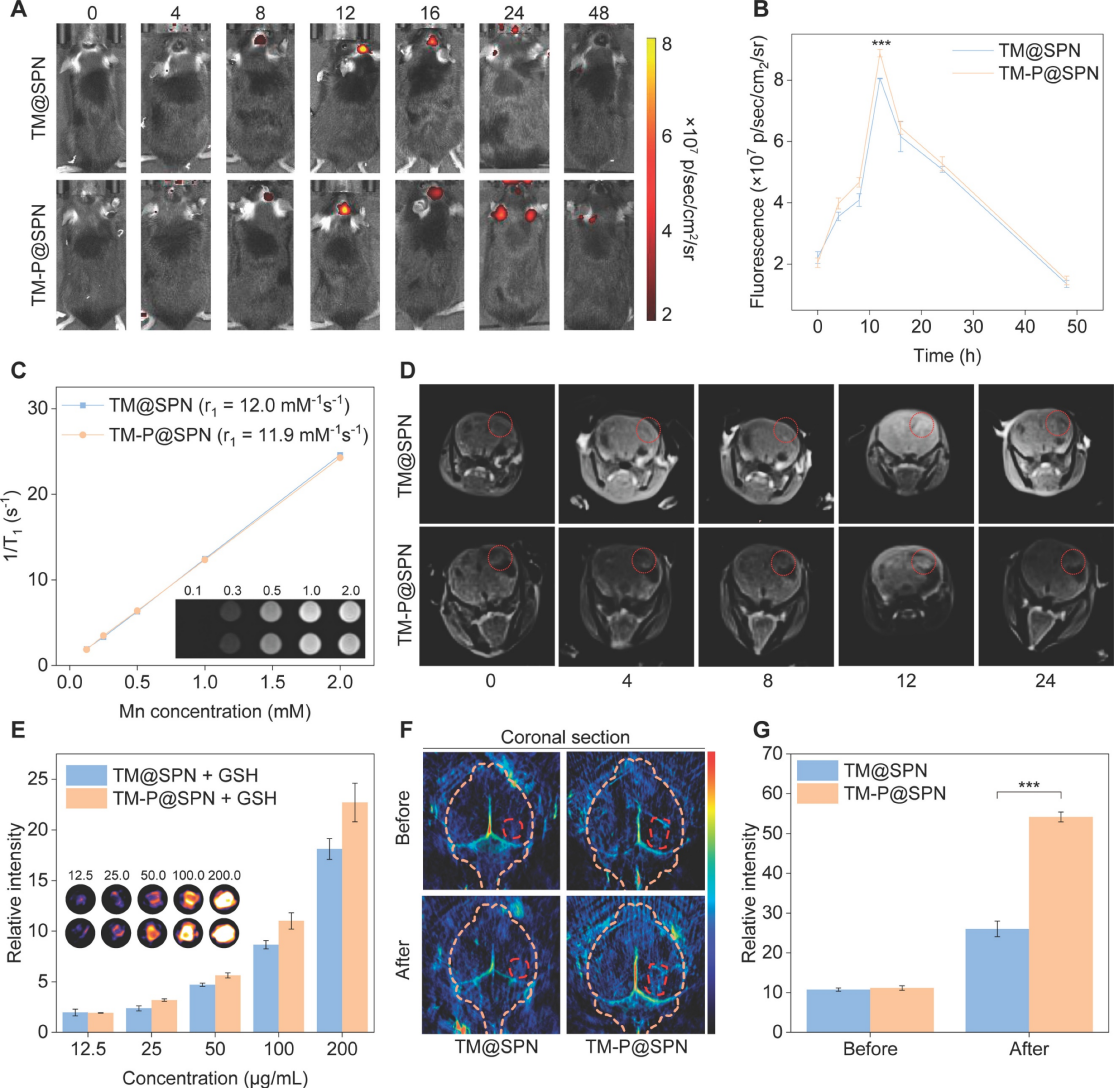
Multimodal imaging performance evaluation. (A) Real-time fluorescence tracking of the brain in orthotopic glioma models following intravenous administration of TM@SPN and TM-P@SPN. (B) The fluorescence intensity at the locations of brain in mice *via* injection of TM@SPN and TM-P@SPN (n = 3). (C) *T*_1_-weighted MR image and linear fitting of 1/*T*_1_ of the TM-P@SPN and TM@SPN at different Mn concentrations. (D) Representative MR images of glioma following intravenous administration of the TM@SPN and TM-P@SPN at different time points. (E) PA pseudocolor images and relative intensity of the TM@SPN and TM-P@SPN at different SP concentrations (n = 3). Representative PA images (F) and corresponding relative intensity (G) of glioma before and 12 h after intravenous injection of TM@SPN and TM-P@SPN (n = 3). Data presented as mean ± SD.

Capitalizing on transferrin's ability to cross the BBB, 52, 53 the *in vivo* imaging performance of the nanochanger TM-P@SPN and its control TM@SPN was investigated and compared in an orthotopic glioma model. Firstly, Figure 4A-B illustrate the temporal changes in fluorescence intensity of the tumor-bear brain following the intravenous injection of TM-P@SPN or TM@SPN. Quantitative fluorescence analysis revealed a temporal profile with maximal signal intensity at 12 h post-administration, indicative of BBB-transgressing capability of TM@SPN and TM-P@SPN. Compared to TM@SPN, the TM-P@SPN exhibited more accumulations in brain locations. Besides, the *in vivo* imaging demonstrated significantly stronger fluorescence intensity in the TM-P@SPN group compared to TM@SPN in brain (p < 0.001). This enhanced imaging performance can be attributed to the GSH-responsive disassembly and subsequent aggregation of TM-P@SPN within the TME. The SP-KLVFF-mediated hydrophobic aggregation after the nanochanger disassembly amplified fluorescence signal. Subsequently, in order to characterize their metabolic behavior, major organs including the heart, liver, spleen, lungs, kidneys, and brain were harvested for biodistribution studies at 12 h after intravenous injections. As revealed in Figure S24, hepatic dominance in fluorescence signals demonstrated hepatic clearance as their primary metabolic pathway in both groups.

Next, the MRI potential of the TM@SPN and TM-P@SPN was evaluated. Initially, we execute *T*_1_-weighted MR phantom studies of TM@SPN and TM-P@SPN. The signal intensity of both TM@SPN and TM-P@SPN increased with the Mn dose. The r_1_ relaxivity was calculated to be 12.0 ± 0.4 mM^-1^s^-1^ for TM@SPN and 11.9 ± 0.4 mM^-1^s^-1^ for TM-P@SPN (Figure 4C). MRI was performed on orthotopic glioma-bearing mice following intravenous administration of TM@SPN or TM-P@SPN. The variation trend of MR signal intensity was consistent with that of FLI, and the intensity reaches the highest peak at 12 h (Figure 4D and Figure S25). These results indicated that both TM@SPN and TM-P@SPN have favourable MRI performance due to the presence of Mn. However, the TM-P@SPN showed superior *in vivo* MR signal intensity compared to TM@SPN under equivalent SP concentrations and same time point, which correlates with its higher Mn loading.

In addition to FLI and MRI, PAI was also performed. As depicted in Figure 4E, both TM@SPN and TM-P@SPN could generate concentration-dependent PA signals in a 10 mM GSH microenvironment, with TM-P@SPN exhibiting stronger intensity than TM@SPN under each concentration, confirming their efficacy as PA contrast agents and the superiority of TM-P@SPN. Whereafter, PAI *in vivo* was explored 12 h post-injection. Compare to TM@SPN, TM-P@SPN demonstrated significantly stronger PA signal intensity at tumor sites (p < 0.001; Figure 4F-G). Thus, it can be seen that GSH-triggered disassembly of TM-P@SPN and subsequent SP-KLVFF-mediated hydrophobic aggregation in the TME provides dual signal amplification - enhancing both fluorescence and PA imaging capabilities.

### Evaluation of antitumor therapeutic efficacy

**Figure 5:**
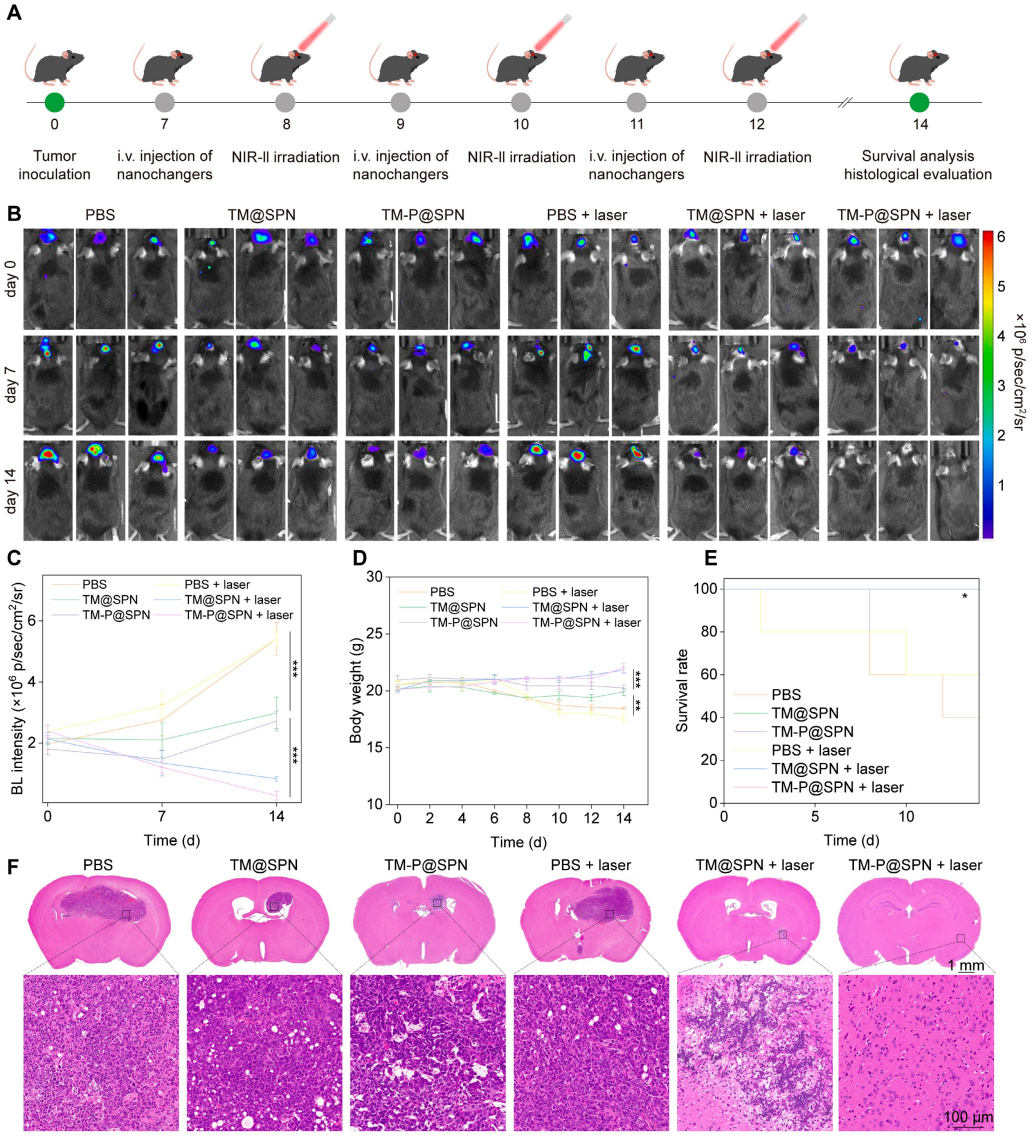
Evaluation of antitumor therapeutic efficacy. (A) Treatment schedule of the nanochangers in combination with laser irradiation. The bioluminescence images (B) and intensity (C) of tumor-bear brain after different treatments on Day 0, 7, and 14. Body weights (D) and survival curves (E) of orthotopic glioma mice during treatment in various groups. (F) Representative H&E staining images of brain sections after various treatments. Data presented as mean ± SD.

Building upon the promising *in vitro* therapeutic outcomes and successful *in vivo* imaging data, the combinatorial treatment efficacy of the nanochangers with laser irradiation were evaluated in an orthotopic glioma model (the detailed therapeutic schedule is presented in Figure 5A). The growth status of the glioma was first observed by the bioluminescence mode in an IVIS Spectrum system (Figure 5B-C). After 14 days of treatments, the combination of TM-P@SPN with laser irradiation (TM-P@SPN + laser) exhibited the weakest bioluminescence signal compared with other groups, suggesting the best antitumor efficiency. By contrast, the effect of all groups followed the order of TM-P@SPN + laser > TM@SPN + laser > TM-P@SPN> TM@SPN > PBS (independent of laser stimulation). Correspondingly, body weight of mice in each group decreased as the therapeutic effect weakened (Figure 5D).

In addition, the survival rate after various treatments was observed and analyzed. As seen in Figure 5E, the group of TM-P@SPN + laser still maintained 100% survival at the 20th day and 80% survival at the 24th day, providing robust evidence of the anti-tumor efficacy. Besides, hematoxylineosin staining (H&E) staining of tumor-bear brain sections was further assessed the therapeutic effects of mice with different treatments. Among all the groups, the area of tumor necrosis in TM-P@SPN + laser group is the smallest, followed by the group of TM@SPN + laser (Figure 5F), which was consistent with the previous conclusion.

### *In vivo* antitumor mechanisms

**Figure 6:**
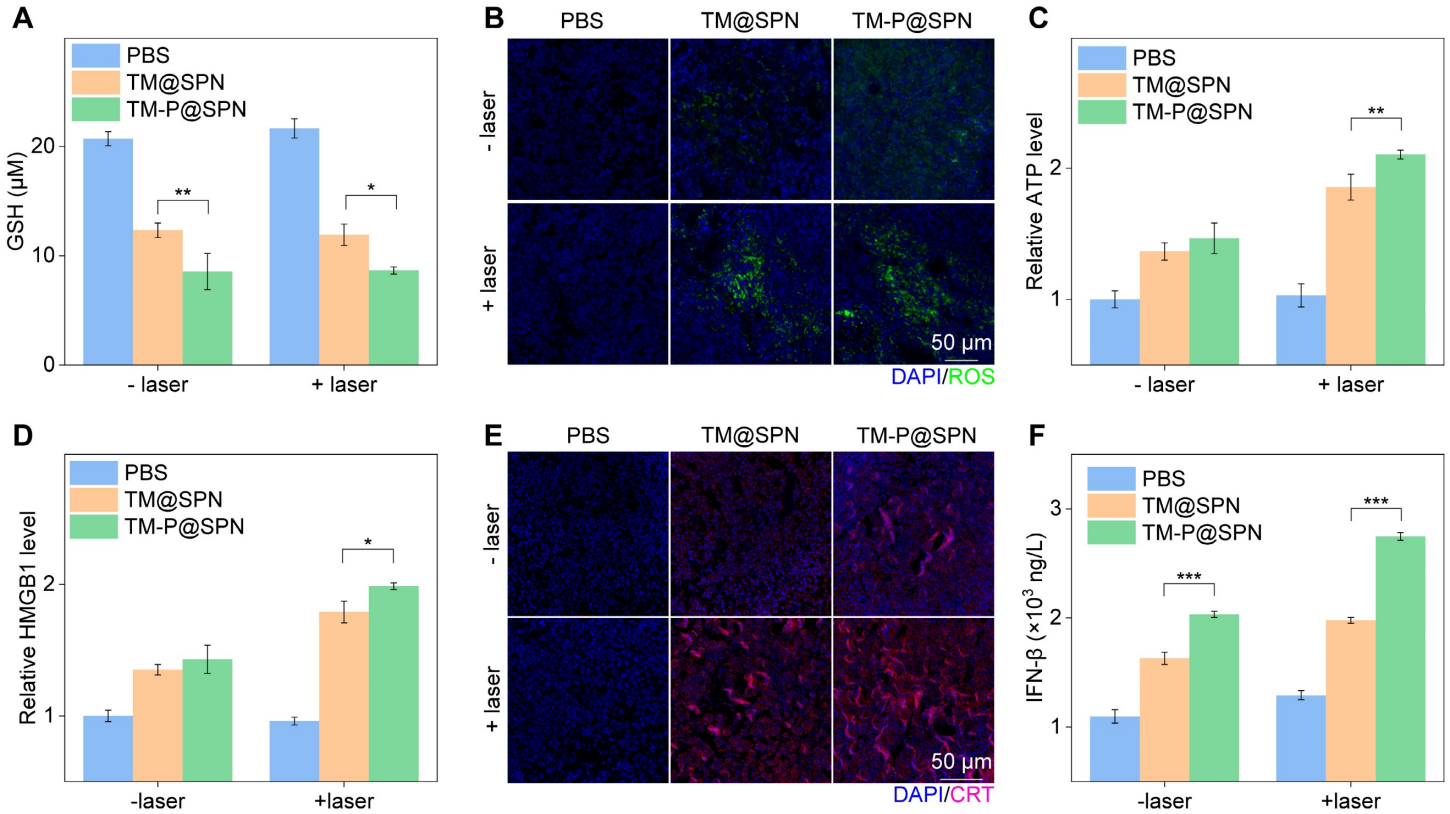
*In vivo* antitumor mechanisms. (A) Intracellular GSH contents in orthotopic glioma models following various treatment regimens (n = 3). (B) Representative green fluorescence images of ROS generation in orthotopic glioma tissue sections following various treatments. Scale bars: 50 μm. (C) The relative ATP levels in tumor tissues after various treatments (n = 3). (D) The relative HMGB1 level in tumor tissues after various treatments (n = 3). (E) Representative immunofluorescence staining images of CRT in tumor tissues after various treatments. Scale bar: 50 μm. (F) IFN-*β* levels in tumor tissues after various treatments (n = 3). Data presented as mean ± SD.

To understand the multiple treatment mechanisms of TM-P@SPN in combination with laser irradiation, some typical index, such as GSH consumption, ROS generation, ATP secretion, HMGB1 release, CRT exposure, and IFN-*β* secretion, were measured in the tumor sites. GSH consumption in tumor sites was first investigated. As shown in Figure 6A, regardless of laser irradiation, GSH consumption was higher in the TM-P@SPN group compared to TM@SPN, suggesting that TM-P@SPN is expected to implement better CDT, thanks to the higher Mn loading rate of TM-P@SPN. Whereas, the highest level of ROS was produced only after TM-P@SPN was combined with laser irradiation (Figure 6B and Figure S26). We consider that laser irradiation elevates ROS levels, an effect further amplified by SP-KLVFF-mediated hydrophobic aggregation within the GSH-riched TME. Whereafter, ICD effect was estimated *in vivo*. Compared to PBS, both TM@SPN and TM-P@SPN significantly enhanced ATP secretion (Figure 6C), HMGB1 release (Figure 6D and Figure S27) and CRT expression (Figure 6E and Figure S28), with particularly pronounced effects observed following laser irradiation. More importantly, the TM-P@SPN combined with laser irradiation demonstrated the most potent ICD effect among all treatment groups. This confirms that SP-KLVFF-mediated hydrophobic aggregation within the GSH-riched TME amplify photothermal signaling, thereby significantly enhancing ICD effects. In addition, IFN-*β* secretion was selected to judge the activated cGAS-STING pathway. As depicted in Figure 6F, TM-P@SPN treatment induced a higher secretion of IFN-*β* than TM@SPN, and the combination of TM-P@SPN and laser irradiation further enhanced IFN-*β* production. These effects were attributed to Mn^2+^-mediated potentiation of the cGAS-STING pathway in tumor-infiltrating DCs 54-56.

### Assessment of immune cell levels *in vivo*

**Figure 7:**
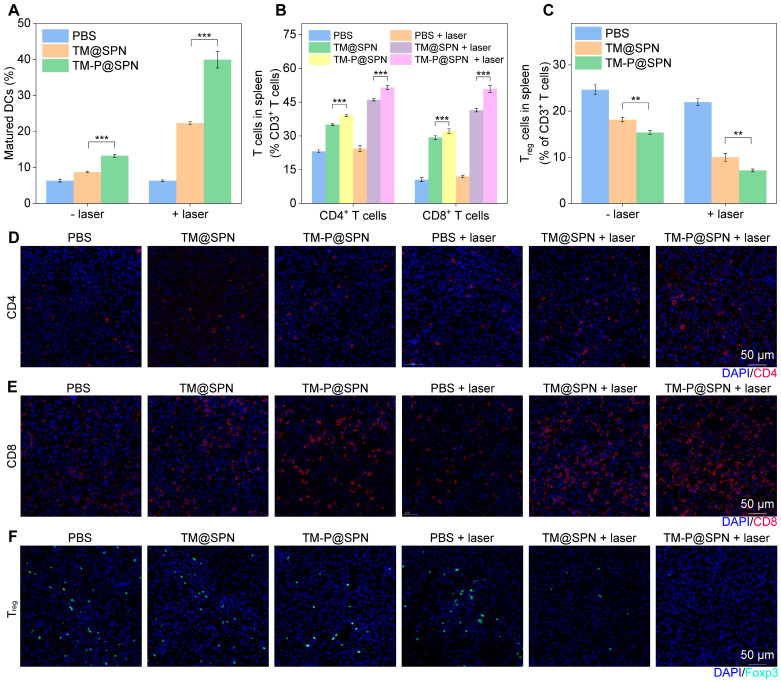
Investigations of* in vivo* immune response. (A) The quantification of CD80^+^ and CD86^+^ DCs extracted from lymph node (n = 3). (B) Quantification of CD4^+^ T cell proportion and CD8^+^ T cell proportion in spleen tissues (n = 3). (C) Quantification of Treg cells proportion in spleen tissues (n = 3). Representative immunofluorescence staining images of tumor-infiltrating CD4^+^ T cells (D), CD8^+^ T cells (E), and Tregs (F) in glioma tumor. Scale bar: 50 μm. Data presented as mean ± SD.

Considering the ICD effect and STING pathway activation that can stimulate DCs maturation and produce immune response, 57, 58 the immune cells from inguinal lymph nodes, spleens, and tumor were collected and analyzed after different treatments. Obviously in lymph nodes, DCs maturation was enhanced through laser irradiation in both TM@SPN and TM-P@SPN groups relative to their non-irradiated counterparts (Figure 7A and Figure S29). The group of TM-P@SPN + laser irradiation showed the highest DCs maturation level compared to other groups. These phenomena may be associated with the induction of ICD and activation of the STING pathway, which could subsequently promote the activation of additional immune cell populations, especially T cells. Consequently, the ratios of spleen-infiltrating CD4^+^、CD8^+^ T cells and regulatory T cells (Tregs) were tested by flow cytometry. As depicted in Figure 7B and S30-31, both CD4^+^ and CD8^+^ T cell populations exhibited a hierarchical ordering, with TM-P@SPN + laser > TM@SPN + laser > TM-P@SPN > TM@SPN > PBS + laser irradiation > PBS. Furthermore, the proportion of Tregs (CD25^+^Foxp3^+^) infiltration in spleen followed an opposite trend, demonstrating the most significant inhibition of immunosuppressive T cells following treatment with TM-P@SPN + laser (Figure 7C and Figure S32).

Immunofluorescence staining of CD4^+^ and CD8^+^ T cells in the TME was also systematically analyzed. Figure 7D-E and S33 showed that the combination therapy of TM-P@SPN + laser induces a significantly higher infiltration of cytotoxic CD4^+^CD8^+^ T cells, followed by TM@SPN + laser. For Tregs in tumor sites, the group of TM-P@SPN + laser exhibited the lowest level of infiltration compared to all other groups (Figure 7F and Figure S34). In a word, TM-P@SPN in combination with laser exposure demonstrated optimal theranostics efficacy, showing superior tumor suppression and the most potent immune activation among all treatment groups, while TM@SPN with laser irradiation showed secondary therapeutic effects. These results proved that the prepared nanochangers can be successfully applied for fluorescence-PA-MR imaging and PTT-CDT-immunotherapy. More importantly, the TM-P@SPN undergoes GSH-responsive disassembles, releasing PEG and enabling hydrophobic SP-KLVFF aggregation *via* hydrogen bonding. This aggregation phenomenon amplifies imaging and therapeutic signals, triggering self-programable theranostics of orthotopic glioma.

Additionally, H&E staining was performed on the major organs (heart, liver, spleen, lung, and kidney) to evaluate the biocompatibility of TM-P@SPN in combination with laser irradiation. There were no apparent morphological changes, necrosis, or inflammatory infiltrates observed in any organ when compared to the group PBS plus laser irradiation (Figure S35). These indicated that the TM-P@SPN plus laser irradiation exhibit negligible *in vivo* toxicity to the mice. To further assess systemic toxicity, complete blood panel and blood biochemical analyses were conducted (Figure S36-S37). Notably, regardless of laser irradiation, both TM-P@SPN and TM@SPN exhibited blood routine parameters and key indicators of liver and kidney function that all remained within normal ranges, showing no statistically significant differences compared to the PBS control group. These results collectively provide compelling evidence for the excellent biosafety and biocompatibility of both TM-P@SPN and TM@SPN with or without laser irradiation, demonstrating their minimal adverse effects on overall systemic homeostasis.

## Conclusion

In summary, we have proposed TME-responsive aggregation strategy through designing a SP-based nanochanger to auto-amplify imaging and therapeutic signals for self-programable theranostics of orthotopic glioma. The P@SPN was initially assembled through chemically stable conjugation of disulfide bond-linked KLVFF-PEG chains with SP, followed by surface modification with TM to form the final nanochanger TM-P@SPN. Owing to TM modification, the TM-P@SPN possess BBB penetration and glioma targeting performance, as well as MRI-guided CDT and STING pathway-activated immunotherapy functions. Critically, upon entering the GSH-rich TME, TM-P@SPN undergoes disulfide cleavage-mediated PEG release and subsequent hydrophobic SP-KLVFF aggregates through hydrogen bonding. This KLVFF-mediated structural transformation endowed TM-P@SPN with superior imaging contrast and photothermal conversion efficiency compared to the control TM@SPN without KLVFF. Consequently, TM-P@SPN demonstrated augmented FLI, enhanced PAI, improved PTT, and amplified ICD effects. Unlike traditional nanotheranostics, this study proposes an intelligent aggregation strategy specifically tailored for implementation at the tumor site to enhance cancer theranostics. The developed TM-P@SPN provides a paradigm for the design of next-generation therapeutic agents capable of microenvironment responsive aggregation, and enhances tumor ablation and imaging fidelity through integrating BBB penetration, tumor-targeted aggregation, multimodal imaging (fluorescence/MR/PA), and synergistic PTT/CDT/immunotherapy. The self-programable theranostics stratagem has brought new expect for the cure of glioma.

## Supplementary Material

Supplementary methods and figures.
