# Supplementary material for: Tumor microenvironment-triggered aggregation of semiconducting polymer nanochangers for self-programable theranostics of orthotopic glioma
Source: Theranostics. 2026 Jan 1;16(2):794–809. doi: 10.7150/thno.123074 (PMC12674931; doi:10.7150/thno.123074)
Supplement: Supplementary file 1 — Supplementary methods and figures. [file thnov16p0794s1.pdf]

Supporting Information

Tumor microenvironment-triggered aggregation of semiconducting polymer nanochangers for self-programable theranostics of orthotopic glioma

Zheming Song,[1]*‡ Li Li,*[2]*‡ Anni Zhu,*[1] *Danling Cheng,*[1] *Yuyun Zhong,*[4] *Zhiming Zheng,*[3]** Cong Song,*[3]** Jingchao Li,*[1]** Zhong Guo*[4]***

[1] State Key Laboratory of Advanced Fiber Materials, College of Biological Science and Medical Engineering, Donghua University, Shanghai 201620, China

[2] The Second Affiliated Hospital of Kunming Medical University, Key Laboratory of Neurological and Psychiatric Disease Research of Yunnan Province, Kunming 650223, China

[3] Shandong Provincial Hospital, Medical Science and Technology Innovation Center, Shandong First Medical University & Shandong Academy of Medical Sciences, Jinan 250117, China

[4] Center for Biological Science and Technology, Faculty of Arts and Sciences, Beijing Normal University, Zhuhai 519087, China

‡ These authors contributed equally to this work.

* Corresponding authors.

E-mail addresses: zhiming0119@126.com (Z. Zheng), songcong@sdfmu.edu.cn (C. Song), jcli.@dhu.edu.cn (J. Li), and z.guo@bnu.edu.cn (Z. Guo)

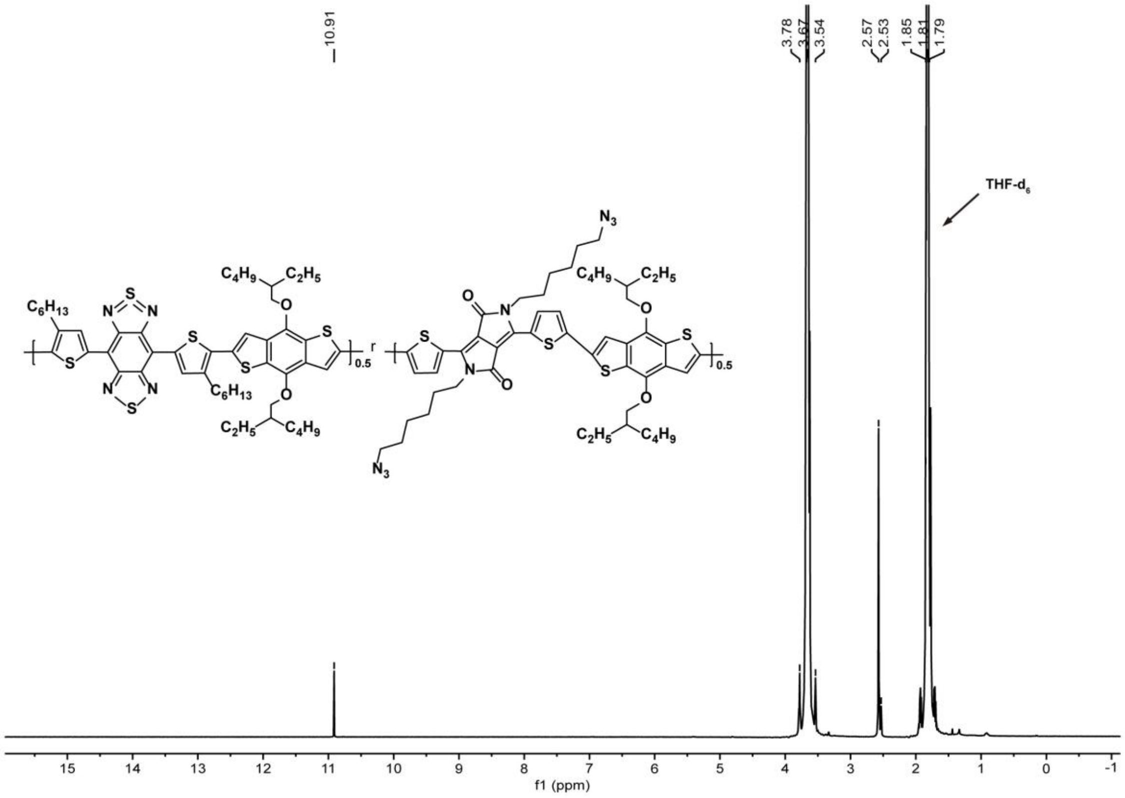

Figure S1. ^{1}H NMR spectrum of SP-N_3.

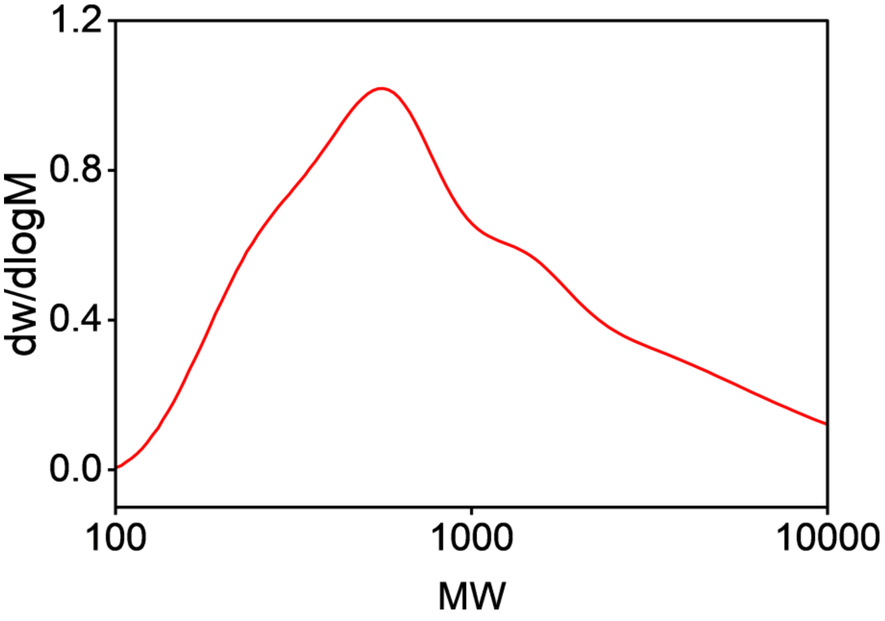

Figure S2. GPC trace of the SP-N_3.

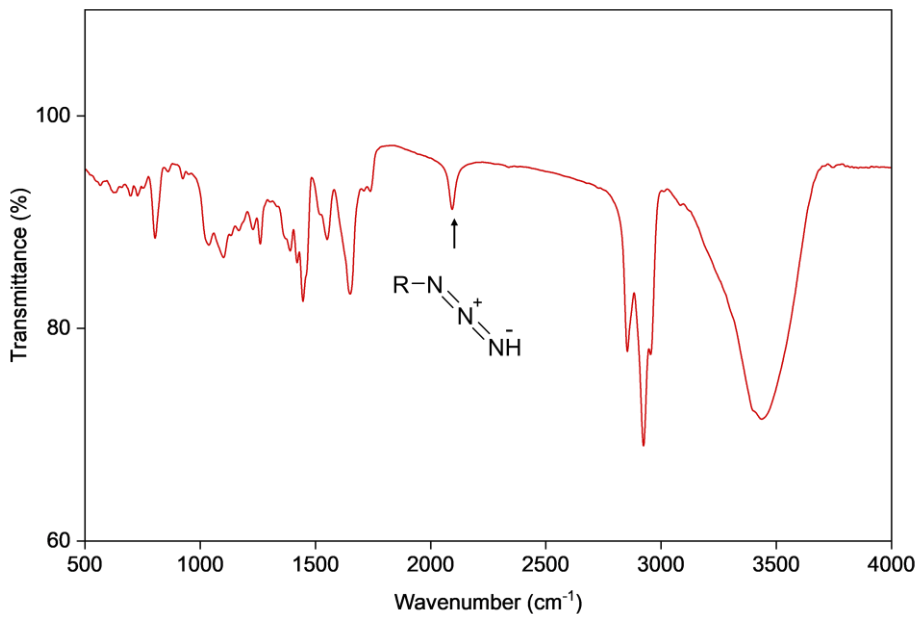

Figure S3. FT-IR spectrum of the SP-N_3.

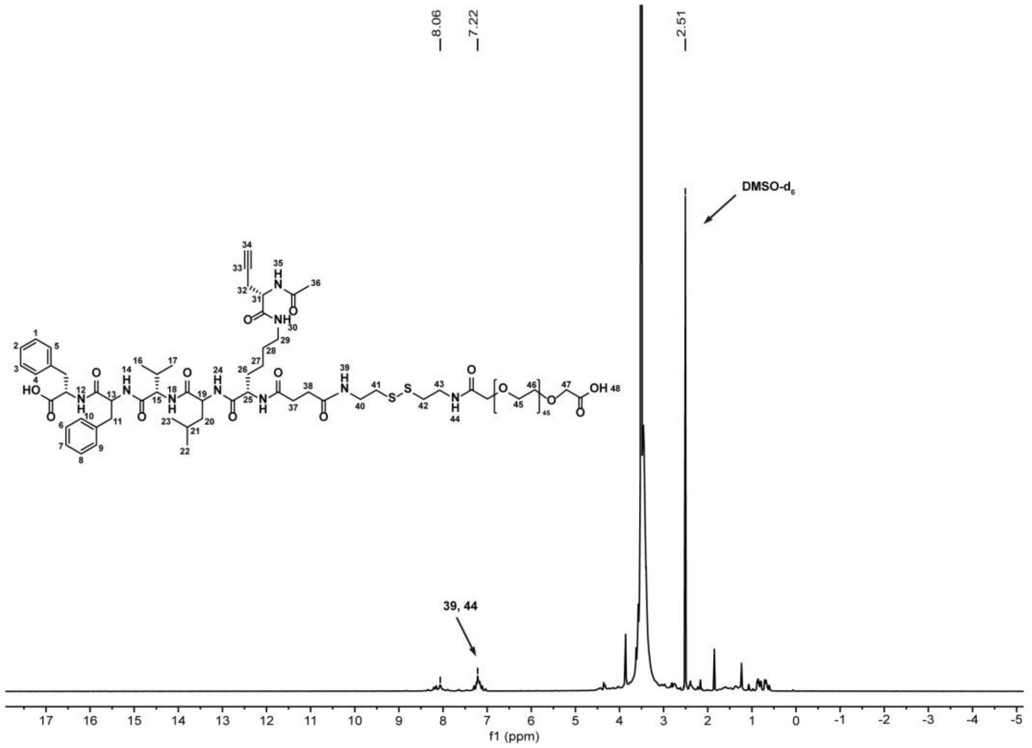

Figure S4. ^{1}H NMR spectrum of KLVFF-PEG.

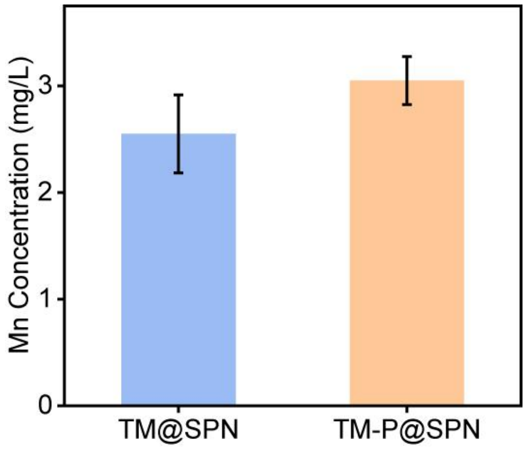

Figure S5. Concentration of Mn in 25 mg/L of TM@SPN and TM-P@SPN solutions (n = 3).

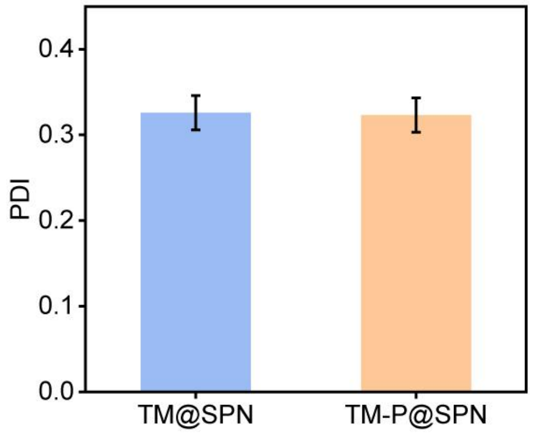

Figure S6. Polydispersity index (PDI) of TM@SPN and TM-P@SPN in aqueous solutions (n = 3).

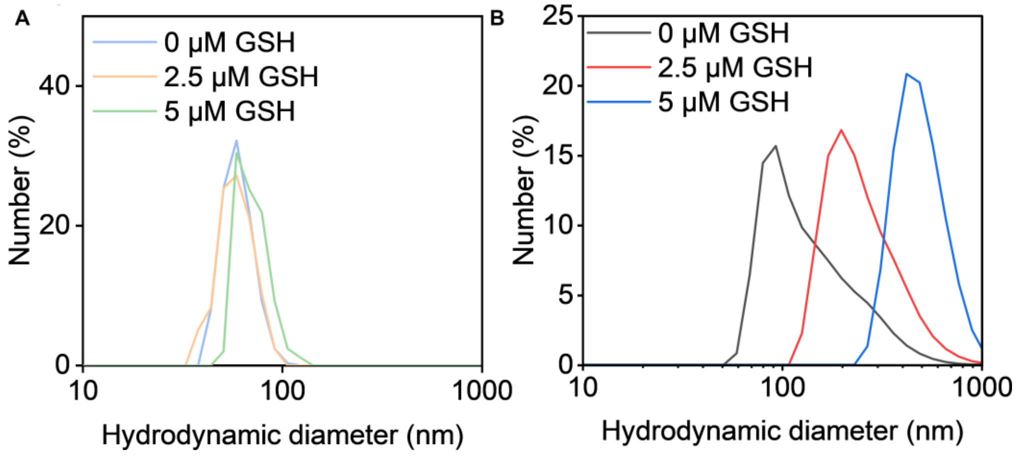

Figure S7. Hydrodynamic sizes of TM@SPN (A) and TM-P@SPN (B) under different GSH-containing solutions.

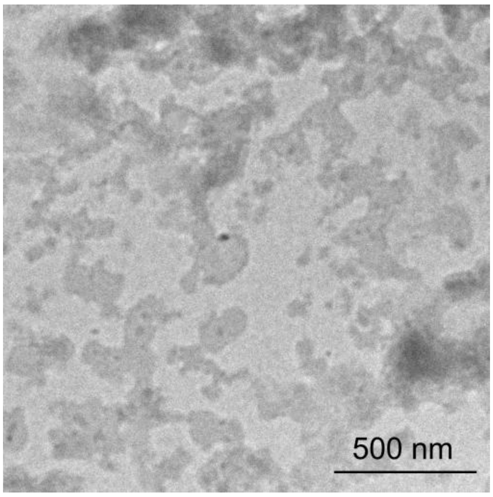

Figure S8. TEM images of TM-P@SPN after GSH-induced aggregation.

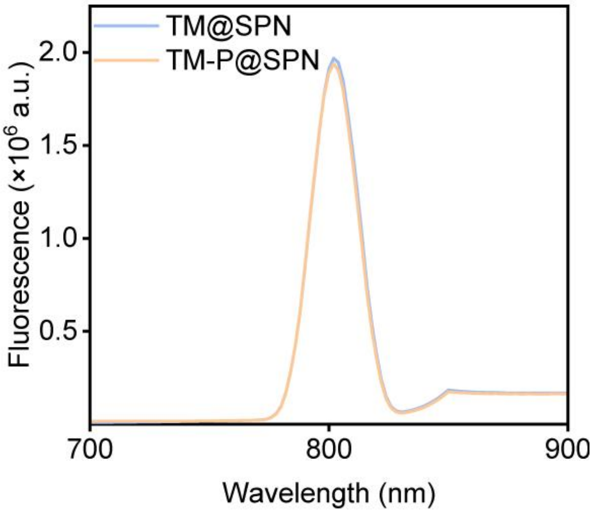

Figure S9. Fluorescence spectra of TM-P@SPN and TM@SPN.

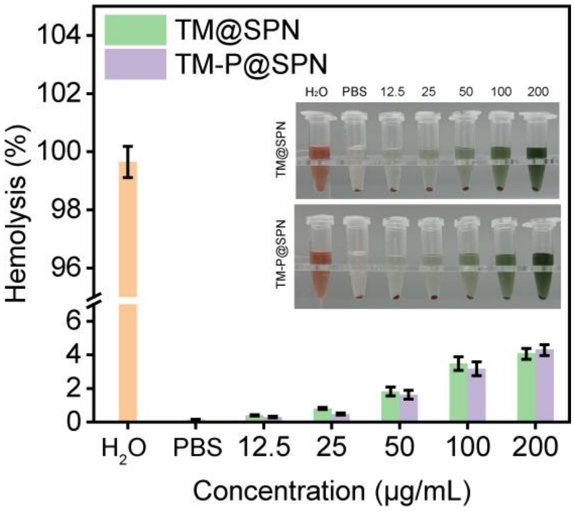

Figure S10. Hemolysis rates and photographs of erythrocytes incubated with different groups (n = 3).

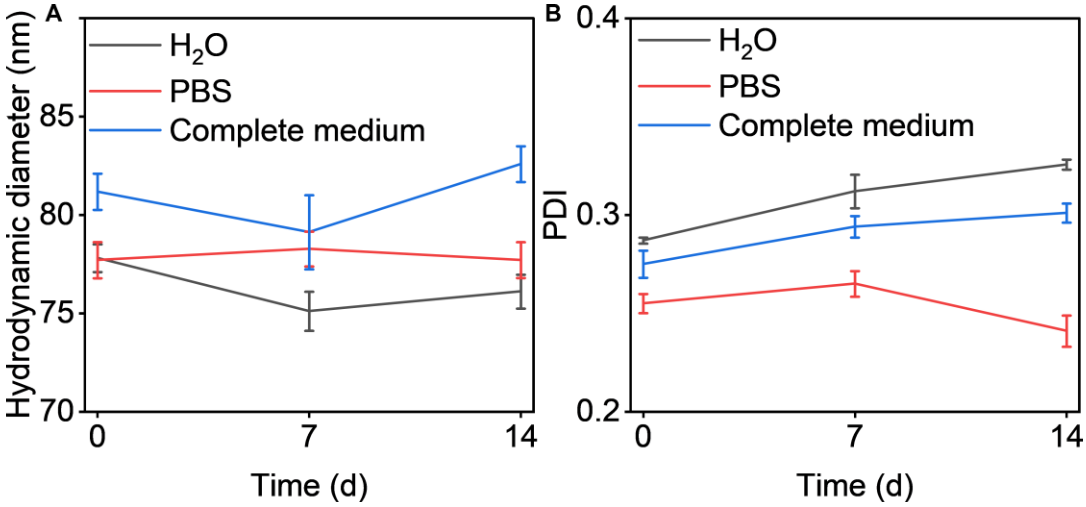

Figure S11. Hydrodynamic diameter (A) and PDI (B) changes of TM-P@SPN over a 14-day period (n = 3).

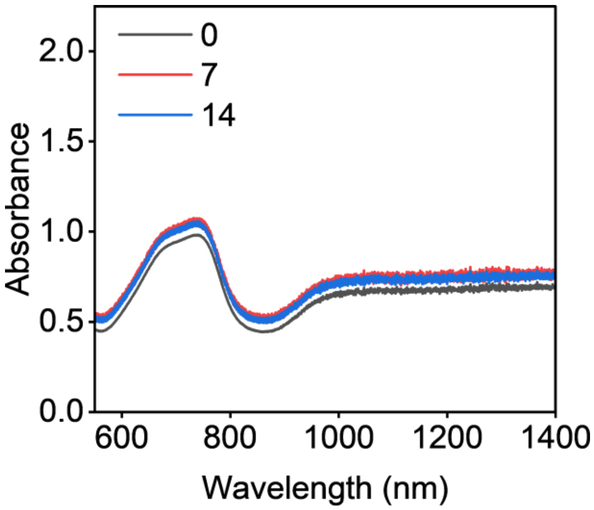

Figure S12. Representative UV-Vis-NIR absorption spectra of TM-P@SPN over a 14-day period.

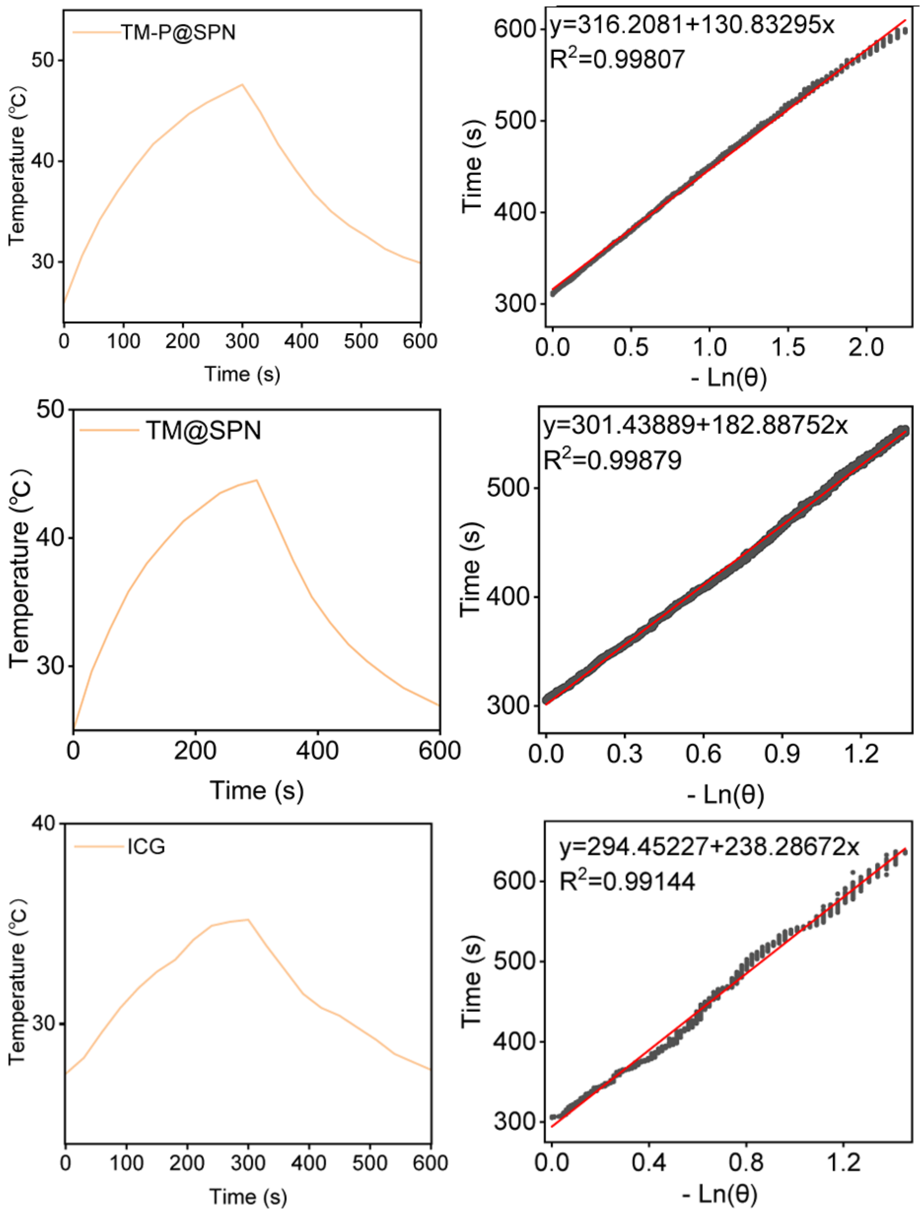

Figure S13. Temperature change curves and cooling time versus –ln θ plots for TM-P@SPN, TM@SPN, and ICG solutions under 1064 nm laser irradiation.

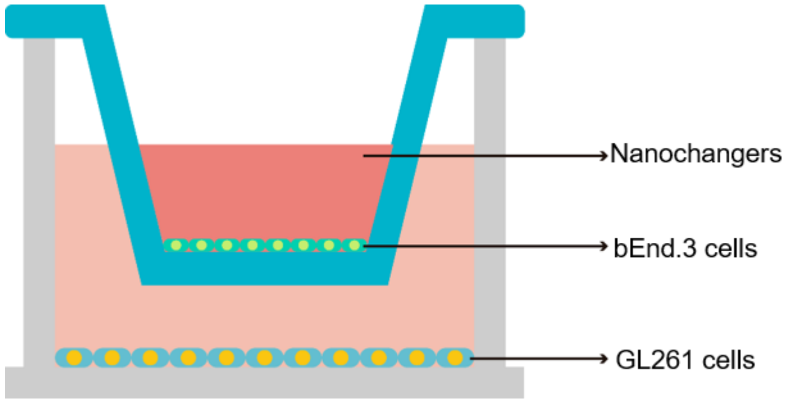

Figure S14. Schematic illustration of the transwell model that simulates the BBB.

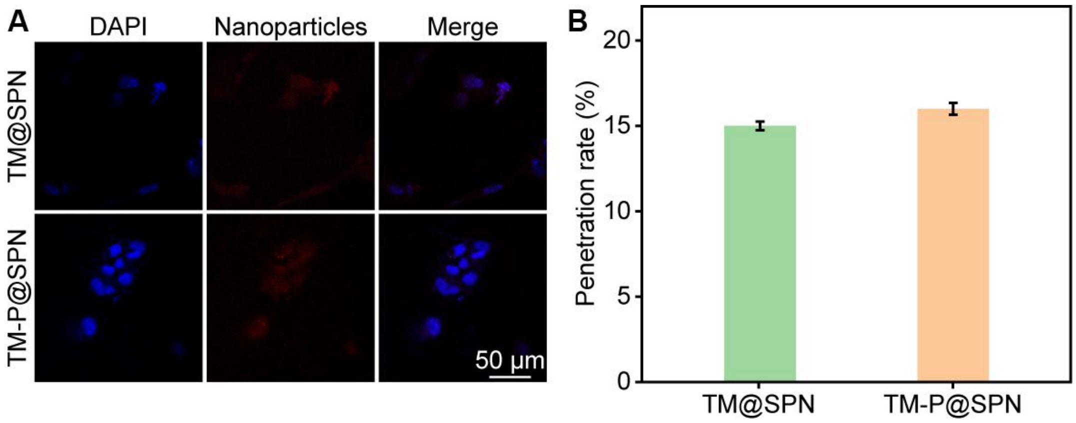

Figure S15. (A) Representative CLSM images in GL261 cells following the uptake of TM@SPN or TM-P@SPN after crossing the bEnd.3 monolayer. (B) The penetration rate of TM@SPN or TM-P@SPN after crossing the bEnd.3 monolayer (n = 3).

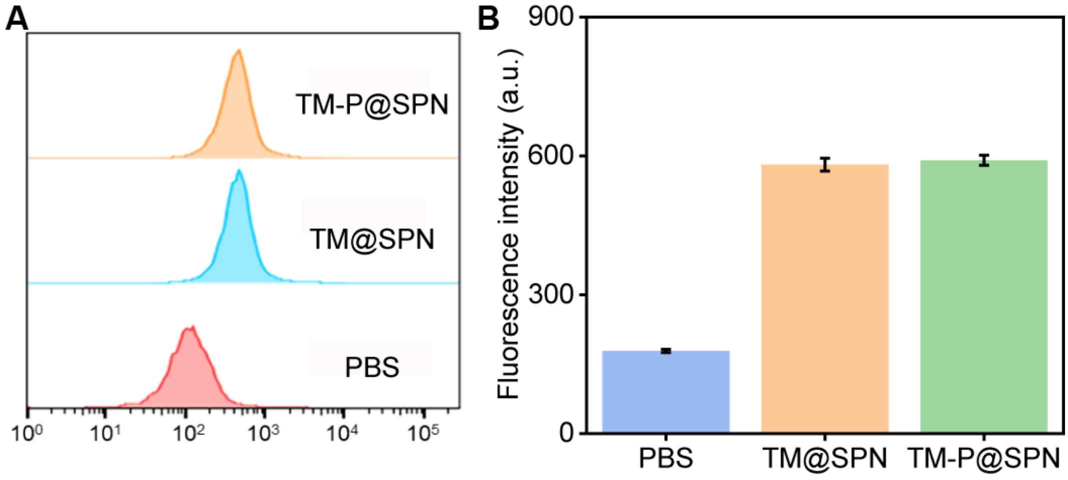

Figure S16. Flow cytometry analysis (A) and quantitative mean fluorescence intensity (B) of GL261 cells treated with PBS, TM@SPN and TM-P@SPN for 6 h (n = 3).

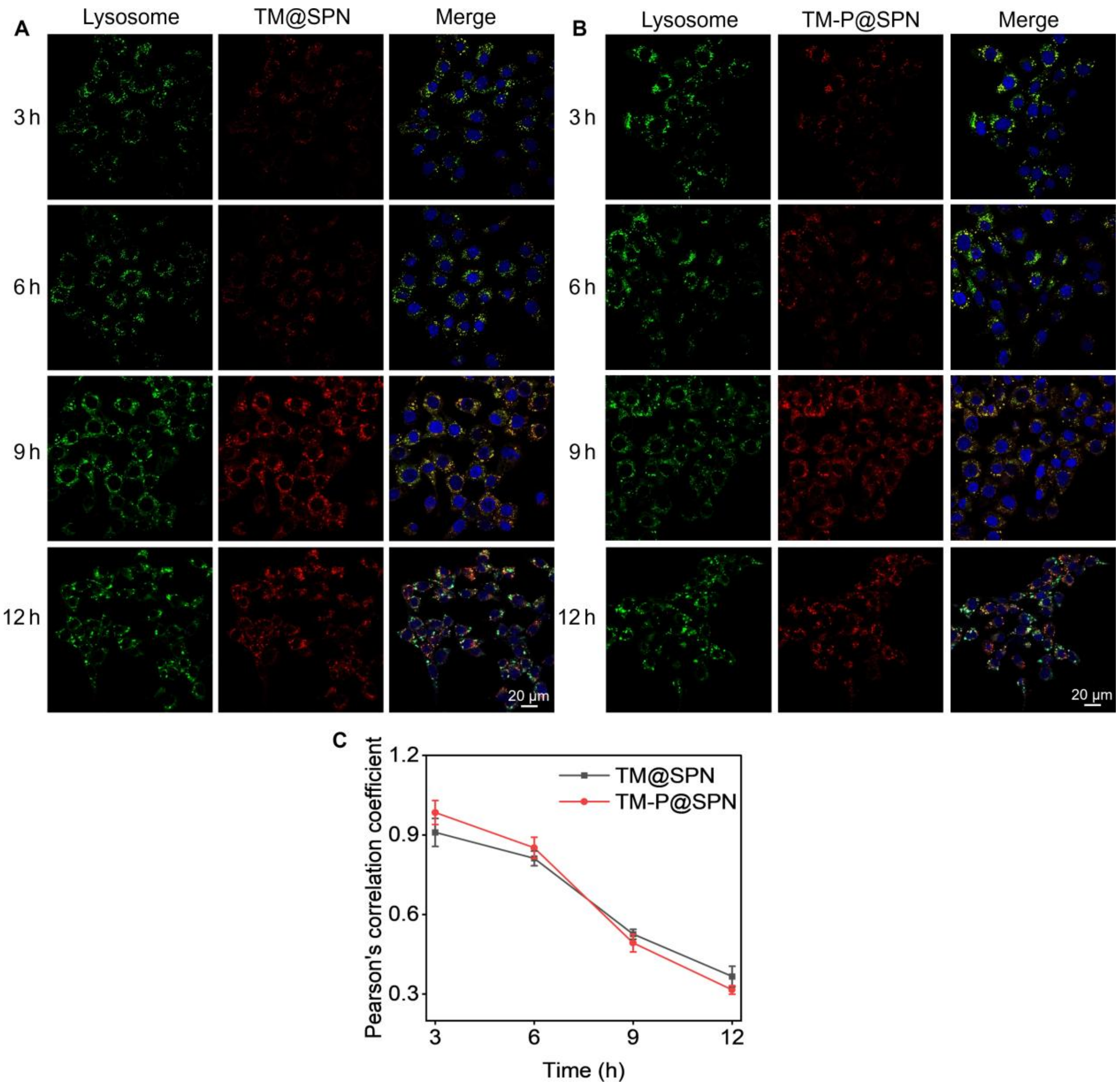

Figure S17. CLSM images of cellular uptake and co-localization of TM@SPN (A) and TM-P@SPN (B) in GL261 cells. Blue: nuclei labeled with DAPI; red: TM@SPN and TM-P@SPN; green: lysosomes labeled with LysoTracker. (C) PCC analysis of co-localization between green and red fluorescence by ImageJ (n = 3).

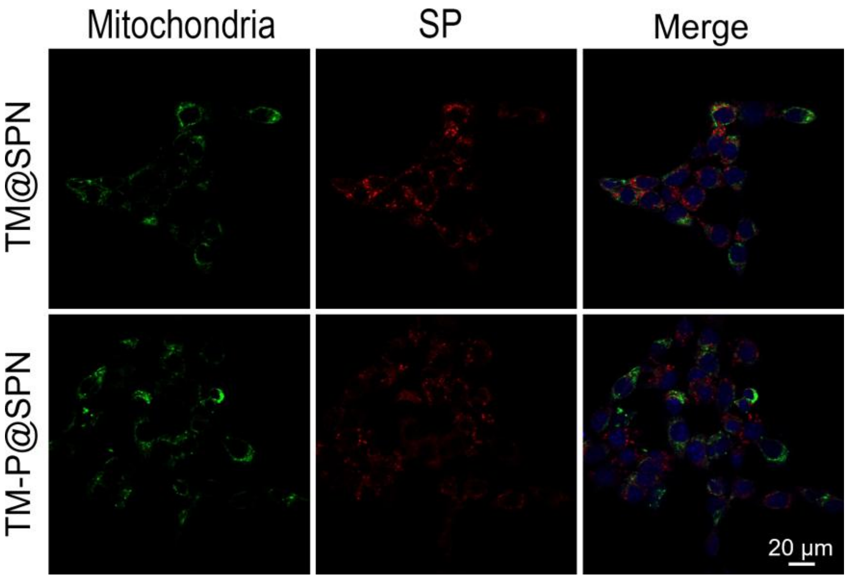

Figure S18. CLSM images of intracellular distribution and mitochondrial co-localization of TM@SPN and TM-P@SPN in GL261 cells. Blue: nuclei labeled with DAPI; red: TM@SPN and TM-P@SPN; green: mitochondria labeled with MitoTracker.

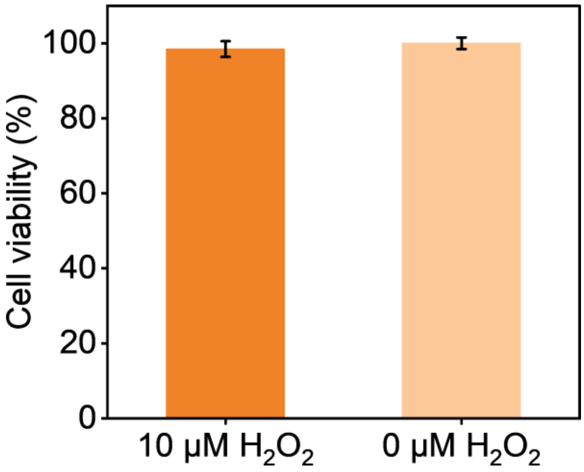

Figure S19. Cell Viability of GL261 cells treated with 100 μM H_2O_2.

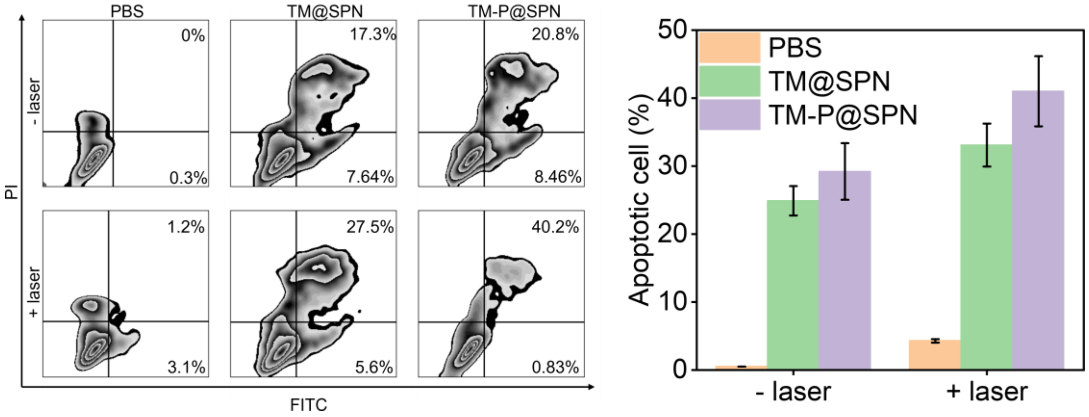

Figure S20. Flow cytometry and quantitative analysis of GL261 cell apoptosis after different treatments (n = 3).

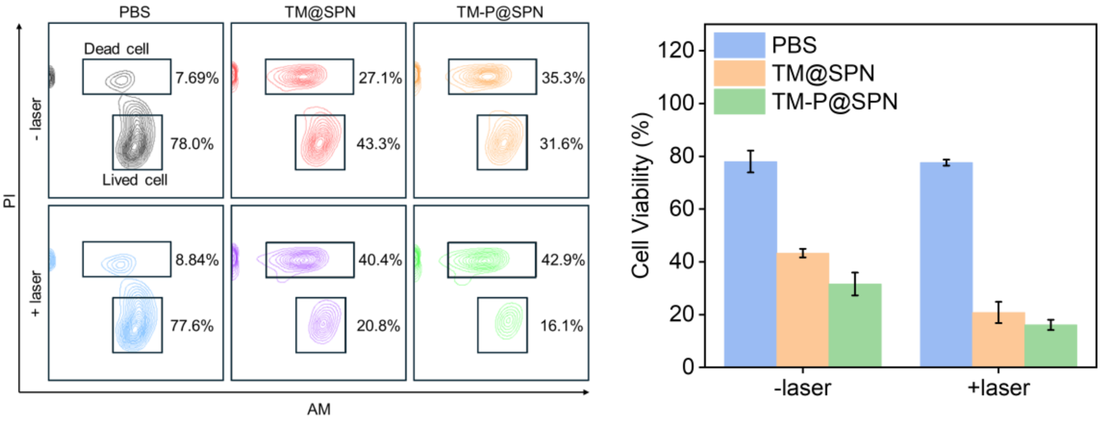

Figure S21. Flow cytometry and quantitative analysis of GL261 cell viability after different treatments using a live/dead staining assay (n = 3).

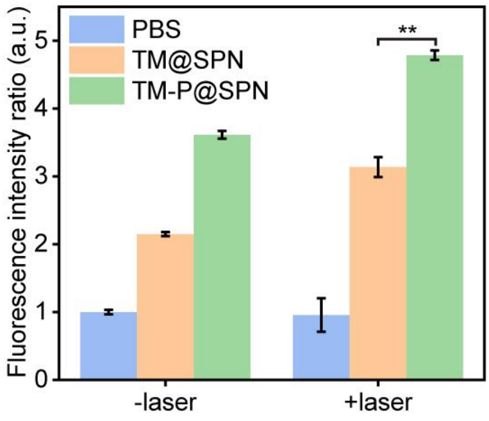

Figure S22. The fluorescence intensity ratio of ROS signals of GL261 cells after different treatments (n = 3).

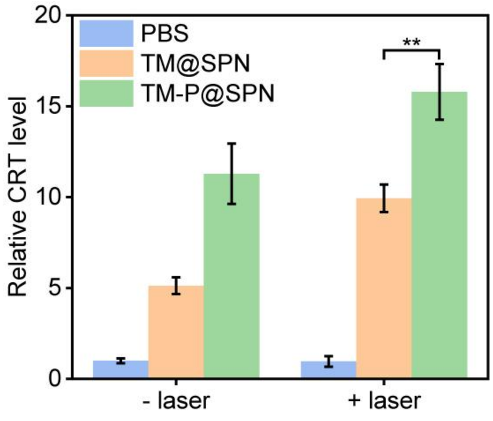

Figure S23. The fluorescence intensity ratio of CRT signals of GL261 cells after different treatments (n = 3).

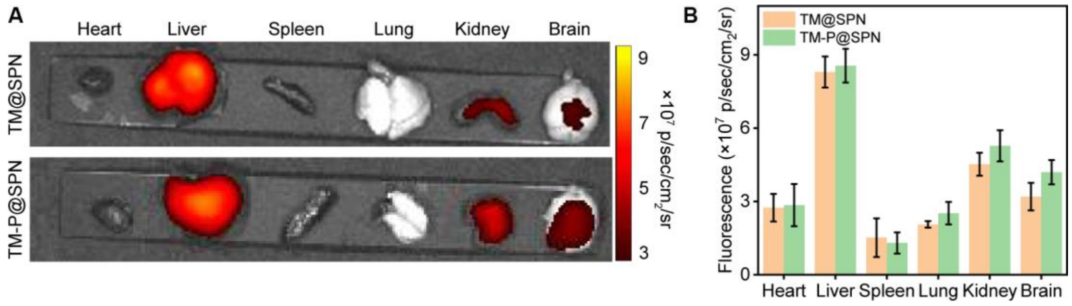

Figure S24. Fluorescence images (A) and intensity (B) of heart, liver, spleen, lung, kidney, and tumor-bearing brain (n = 3).

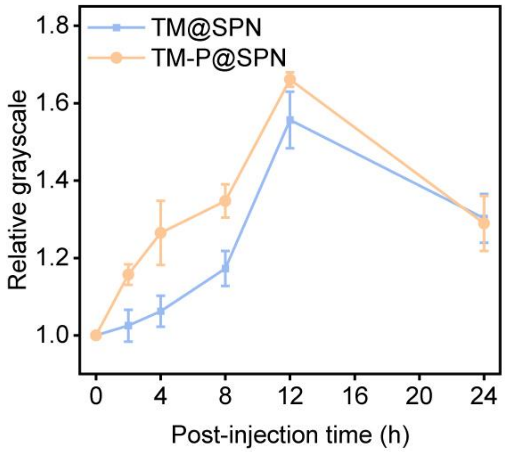

Figure S25. MR relative grayscale of tumor at different time points post intravenous injection of the TM@SPN and TM-P@SPN (n = 3).

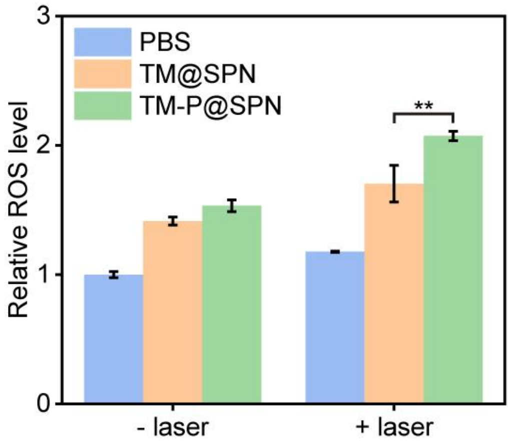

Figure S26. The fluorescence intensity ratio of ROS signals in tumor sites after different treatments (n = 3).

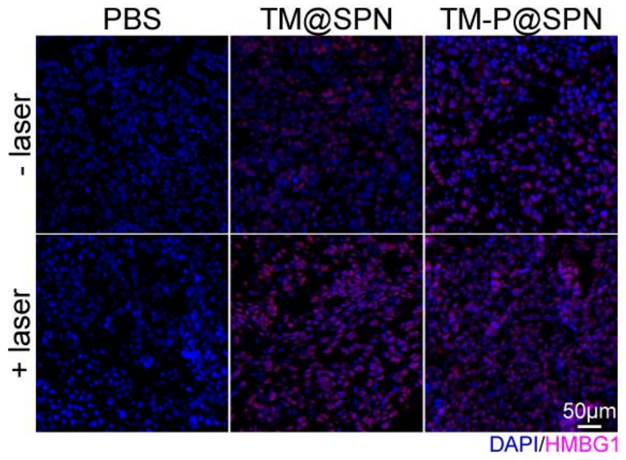

Figure S27. Immunofluorescence staining images of HMGB1 in tumor tissues after different treatments.

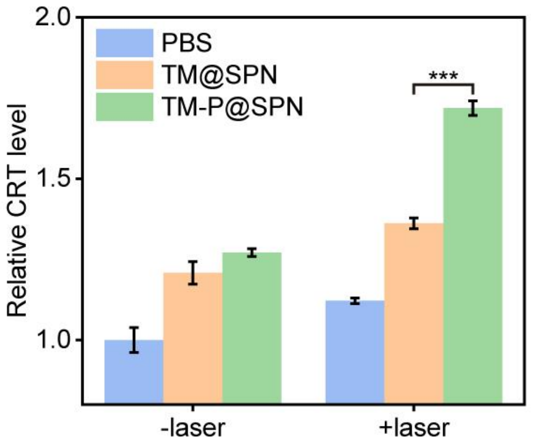

Figure S28. The fluorescence intensity ratio of immunofluorescence staining of CRT in tumor tissues after different treatments (n = 3).

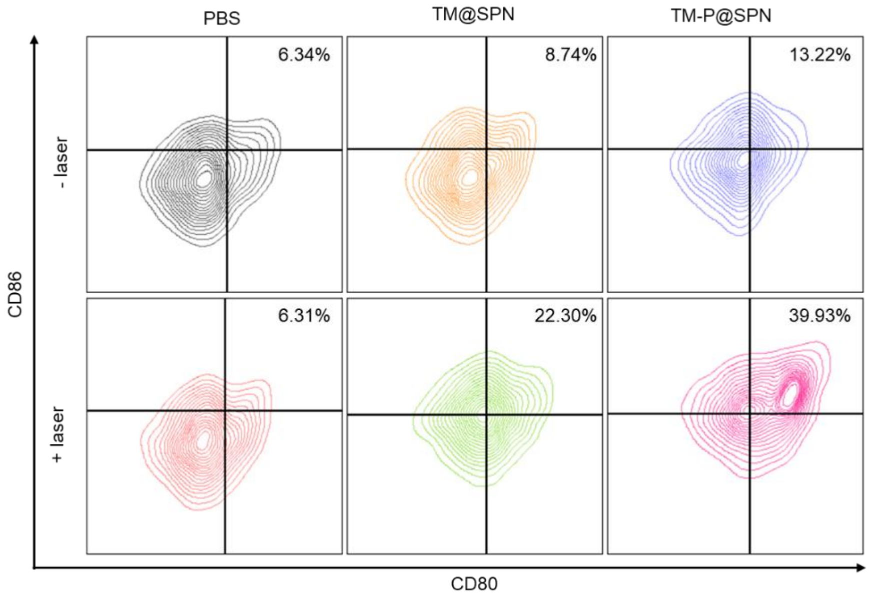

Figure S29. Representative flow cytometry plots of DCs in the lymph nodes after different treatments.

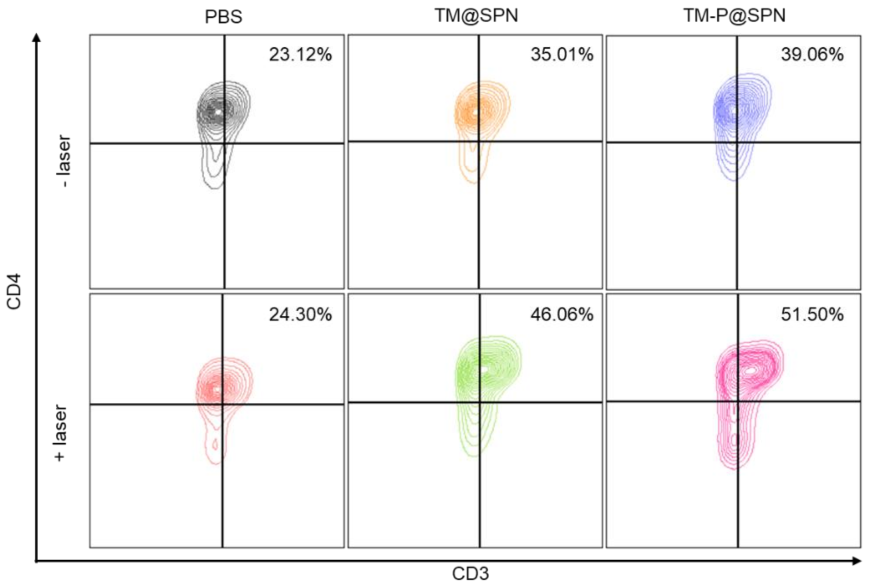

Figure S30. Representative flow cytometry plots of $CD3^{+}CD4^{+}$ T cells in the spleen after different treatments.

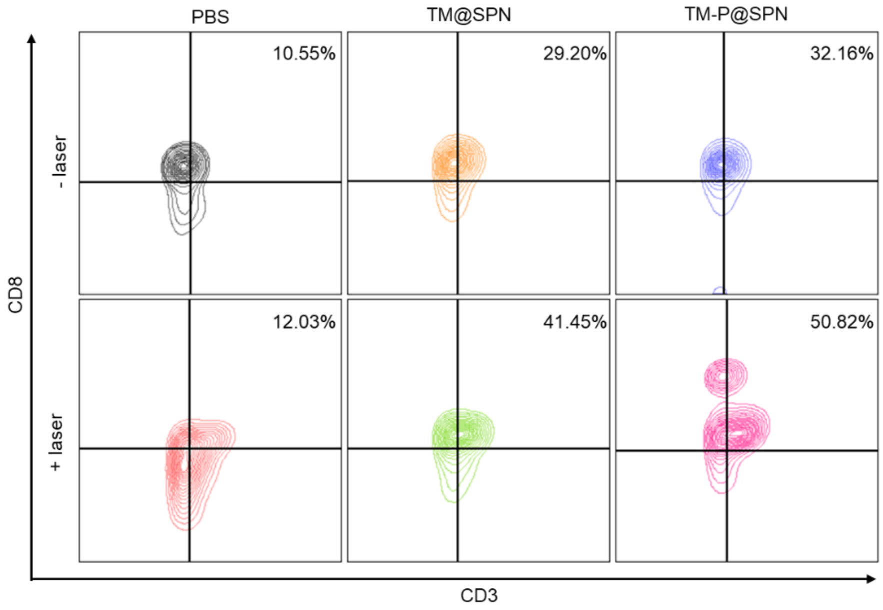

Figure S31. Representative flow cytometry plots of $CD3^{+}CD8^{+}$ T cells in the spleen after different treatments.

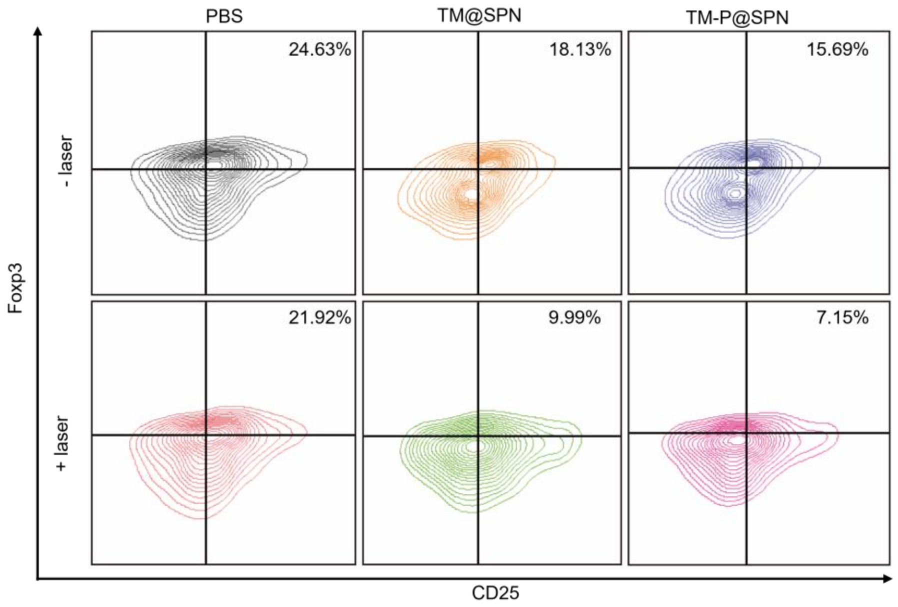

Figure S32. Representative flow cytometry plots of Tregs in the spleen after different treatments.

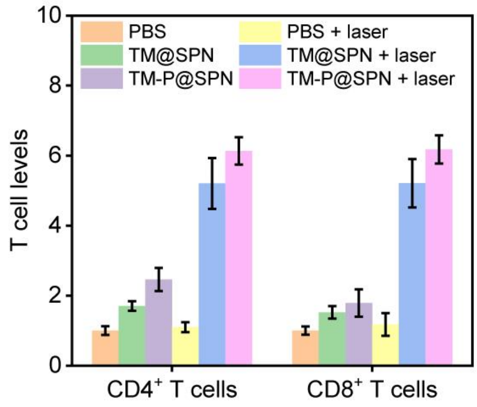

Figure S33. The fluorescence intensity ratio of $CD4^+$ T cells and $CD8^+$ T cells in the tumor after different treatments (n = 3).

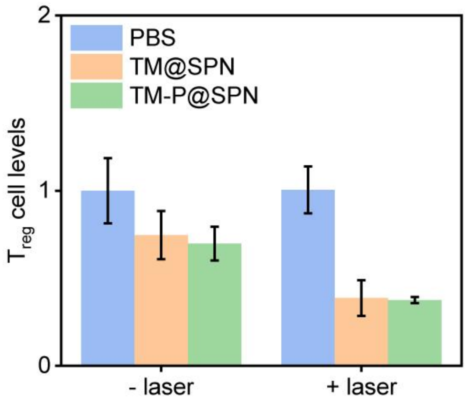

Figure S34. The fluorescence intensity ratio of Tregs in the tumor after different treatments (n = 3).

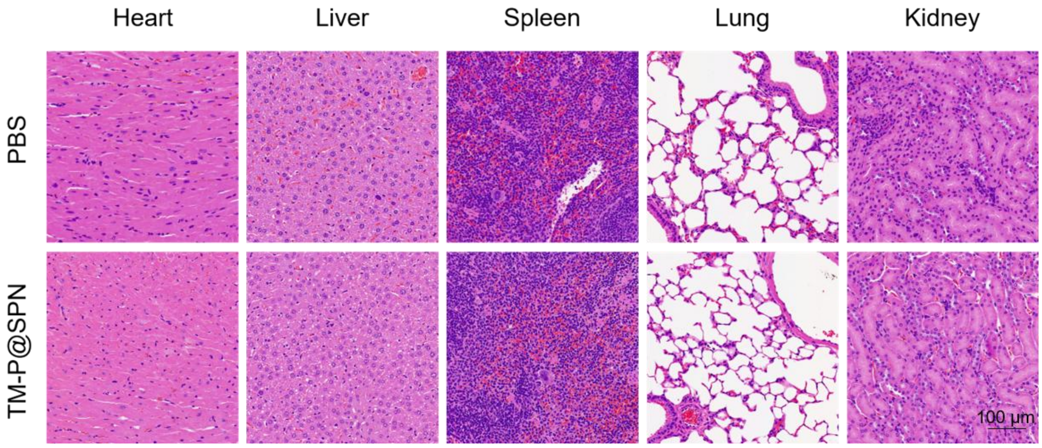

Figure S35. H&E staining of major organs of the group of PBS and TM-P@SPN under the condition of laser irradiation.

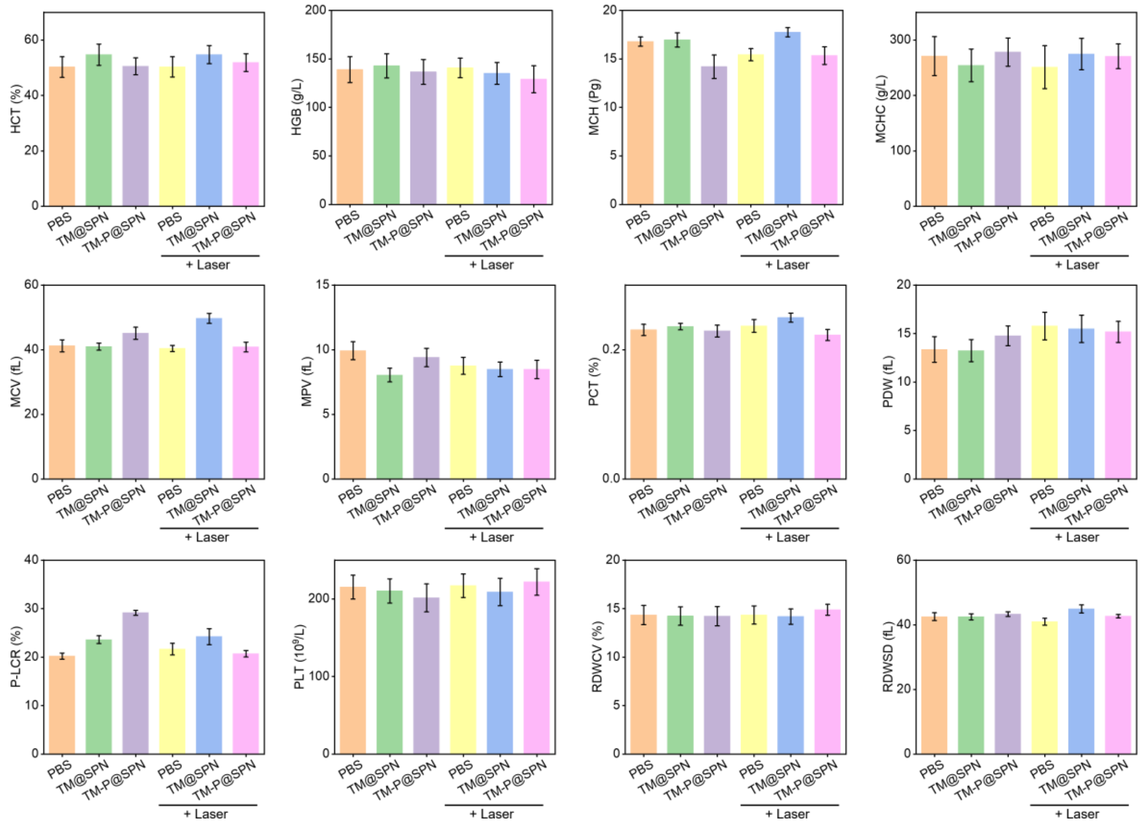

Figure S36. The blood routines from healthy mice in different treatment groups.

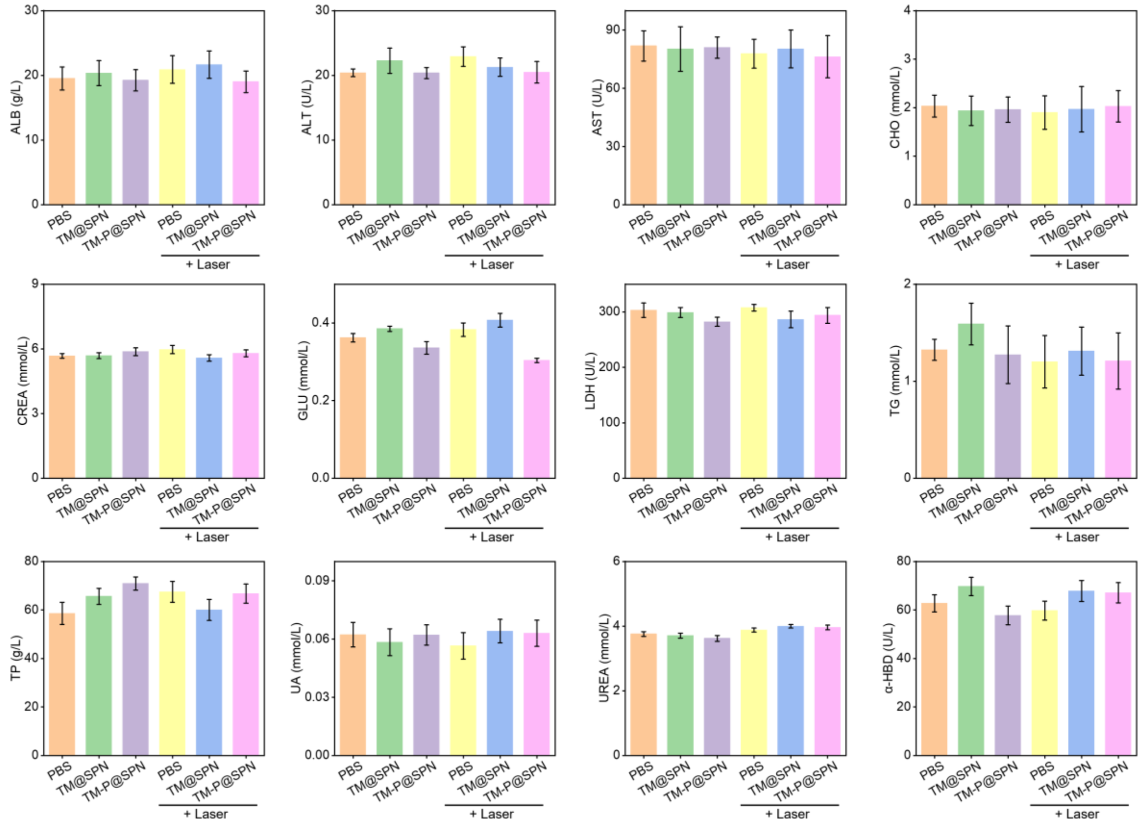

Figure S37. Blood biochemical analysis of healthy mice in different treatment group.

Materials and methods

Materials

The β-amyloid peptide fragment (Lys-Leu-Val-Phe-Phe, KLVFF) was procured from Hangzhou Allpeptide Biotechnology Co., Ltd. (China). Cystamine (NH_2-s-s-NH_2), 1-(3-dimethylaminopropyl)-3-ethylcarbodiimide hydrochloride (EDC·HCl), N-hydroxysuccinimide (NHS), Transferrin (Tf), bovine serum albumin (BSA), pentamethyldiethylenetriamine (PMDETA), reduced glutathione (GSH), and methylene blue (MB) were sourced from Sigma-Aldrich (USA). COOH-PEG-COOH (MW: 2000 Da) was obtained from Xi'an Ruixi Biological Technology Co., Ltd. (China). Alkynyl-PEG-COOH (MW: 2000 Da) and copper(I) bromide (CuBr, 99.9%) were acquired from Macklin Biochemical Co., Ltd. (China). Cell culture reagents, including DMEM and FBS, were purchased from Thermo Fisher Scientific (China). Antibodies for flow cytometry and immunohistochemistry were supplied by BioLegend (USA) and Abcam (UK). Commercial assay kits for biochemical analyses were provided by Beyotime Biotechnology (China) and R&D Systems (USA). All organic solvents were obtained from Sinopharm Chemical Reagent Co., Ltd. (China).

General characterization techniques

^{1}H NMR spectra were recorded on a Bruker AV 400 NMR spectrometer (Karlsruhe, Germany). All samples were dissolved in DMSO-d6 before measurements. Gel permeation chromatography (GPC) analysis was performed on an Agilent 1260 Infinity II system (Agilent Technologies, USA) equipped with a refractive index detector. Samples were dissolved in tetrahydrofuran (THF) at a concentration of 2 mg/mL and filtered through a 0.22 μm PTFE membrane prior to injection. Fourier transform infrared (FTIR) spectrum was recorded on a Nicolet 6700 FTIR spectrophotometer (Thermo Fisher Scientific Nicolet iS20, Massachusetts, Waltham). Samples were mixed with milled KBr crystals and pressed to form 13-mm diameter disks before measurements. Zeta potential and dynamic light scattering (DLS) measurements

were performed using a Malvern Zetasizer Nano ZS model ZEN3600 (Worcestershire, UK) coupled with a standard 633-nm laser. Samples were dispersed in phosphate buffer with a concentration of 1 mg/mL before measurements. Transmission electron microscopy (TEM) imaging was executed using a Tecnai G2 F20 TWIN, FEI analytical electron microscope (Oregon, USA) at an operating voltage of 200 kV. Each sample was prepared by dropping an aqueous particle suspension onto a carbon coated copper grid and air dried before measurements. UV-vis spectra were collected using a UV-3600 Shimadzu spectrometer (Kyoto, Japan). Samples were dispersed in water before measurements. The content of manganese (Mn) was checked *via* Leeman Prodigy inductively coupled plasma-optical emission spectroscopy (ICP-OES, Hudson, NH). Samples were dispersed in water before measurements. Fluorescence spectra were measured by RF-6000 fluorescence spectrometer (Shimadzu, Nishinokyo, Japan). Samples were dispersed in water with a concentration of 1 mg/mL before measurements.

Evaluation of hemocompatibility

Hemolysis assay was performed to assess the hemocompatibility of the TM@SPN and TM-P@SPN according to the literature.[1] Briefly, 1.5 mL of blood collected from the inner canthus vein plexus of mice was diluted with 3.5 mL of PBS, and then the pure red blood cells (RBCs) were obtained *via* repeated centrifugation/redispersion steps (2000 rpm, 10 min, 3 times). The RBCs were diluted with 5 mL of PBS. Afterwards, 100 μL of the obtained RBC suspension was mixed with 900 μL PBS (negative control), water (positive control), and PBS solutions of TM@SPN or TM-P@SPN at different concentrations (12.5, 25, 50, 100, or 200 μg/mL), respectively. After 2 h incubation at 37 ^{o}C, all the samples were centrifuged at 10000 rpm for 5 min. Then, the photos of the samples were taken and the absorbance of the obtained supernatant at 540 nm was measured *via* UV-vis spectrometry. The hemolysis rate was calculated according to the following formula: Hemolysis rate (%) = $[(A_{(sample,\ 540\ nm)} - A_{(negative,\ 540\ nm)}) / (A_{(positive,\ 540\ nm)} - A_{(negative,\ 540\ nm)})] \times 100\%$.

Cell culture

The Murine glioma cell line (GL261 cells), murine brain microvascular endothelial cell line (bEnd.3 cells) and murine dendritic cells (DCs) were regularly cultured and passaged using DMEM medium supplemented with 10% FBS and 1% penicillin-streptomycin (medium named DMEM^{++}) at 37 °C in a Thermo Scientific cell incubator (Waltham, MA) with 5% CO_2.

Cytotoxicity assay

Cytotoxicity of TM-P@SPN and TM@SPN was measured *via* cell counting kit (CCK-8) assay of cell viability using a Thermo Scientific Multiskan MK3 ELISA reader (Waltham, MA) at a wavelength of 450 nm. Briefly, GL261 cells were first seeded into a 96-well plate at a density of 5×10^3 cells per well with 0.1 mL DMEM^{++} medium and were cultured overnight in cell incubator. In the next day, the medium of each well was replaced with 100 μL fresh DMEM^{++} medium containing TM-P@SPN or TM@SPN at different SP concentrations (6, 12.5, 25, 50, and 100 μg/mL, respectively). For comparison, 100 μL fresh DMEM^{++} medium was used as a control. After the cells were cultured for 24 h, CCK-8 solution (10 μL) was added to each well and cells were incubated for additional 2 h under regular culture conditions. Finally, the absorbance of each well was quantified to evaluate the cytocompatibility of TM-P@SPN or TM@SPN.

Establishment of the *in vitro* Blood-Brain Barrier (BBB) model

A transwell device was used to establish the *in vitro* BBB model according to the literature.[2] In brief, bEnd.3 cells were seeded in the upper chambers (pore size: 0.4 μm) at 2×10^5 cells/insert. The medium was changed every 2 days. After 6 days, GL261 cells were seeded on the lower chamber. After co-incubation for 24 h, the integrity of the barrier was confirmed by detecting the trans endothelial electrical resistance (TEER) using an epithelial volt-ohmmeter (Millipore, Burlington, VT). Monolayers with TEER over 200 Ω/cm^2 were used for further experiments.

Evaluation of transmembrane ability and cellular uptake

In the *in vitro* BBB model, TM-P@SPN or TM@SPN dispersed in fresh culture medium ([SP] = 25 μg/mL) was added to the upper chamber of transwell device. After co-incubation for 6 h, GL261 cells in the lower chambers were then washed three times with PBS, fixed with 4% glutaraldehyde at 4 °C for 15 min, stained with DAPI for 15 min, washed with PBS for 3 times, and observed by ZEISS confocal laser scanning microscope (CLSM, Jena, Germany) to check their fluorescence signals. The permeability of TM@SPN or TM-P@SPN through the bEnd.3 monolayer was calculated by the ratio of the fluorescence intensity of the added nanochangers to that in GL261 cells.

Cellular uptake assay

According to previous protocol,[2] GL261 cells were seeded in 12-well plates at a density of 1×10^5 cells per well in 1 mL of DMEM^{++} medium and were cultured overnight. After that, the medium of each well was substituted by 1 mL of fresh DMEM^{++} medium containing the TM-P@SPN or TM@SPN at SP concentration of 25 μg/mL for 6 h. GL261 cells were collected and measured by BD FACSCalibur flow cytometer (Franklin Lakes, NJ). Each measurement was repeated for 3 times.

Colocalization experiment

To investigate the intracellular trafficking and organelle localization of the TM-P@SPN and TM@SPN, GL261 cells were seeded in confocal dishes at a density of 2×10^4 cells per dish and cultured overnight in DMEM^{++}. For lysosomal colocalization studies, the cells were then treated with TM@SPN or TM-P@SPN ([SP] = 25 μg/mL) for predetermined time periods (3, 6, 9, and 12 h). LysoTracker Green was added to the culture medium 2 h prior to the end of incubation. For mitochondrial colocalization, the cells were then treated with TM@SPN or TM-P@SPN ([SP] = 25 μg/mL) for 12 h. MitoTracker Green was added to the culture medium 0.5 h prior to the end of incubation. After staining, the cells were washed three times with PBS.

Subsequently, the cells were fixed with 4% paraformaldehyde for 15 min at room temperature and the nuclei were labeled with DAPI for 10 min. The cells were washed twice with PBS and analyzed with CLSM. The lysosomal colocalization analysis was conducted based on Pearson's correlation coefficient (PCC) with ImageJ software.

Effect of CDT combined with PTT *in vitro*

The effect of CDT combined with PTT of TM-P@SPN and TM@SPN *in vitro* was evaluated by CCK-8 assay. Similar to cytotoxicity assay, GL261 cells were first cultured on demand in 96-well plates. Then, the medium of each well was replaced with 100 μL fresh DMEM^{++} medium containing TM-P@SPN or TM@SPN at SP concentration of 25 μg/mL. Meanwhile, hydrogen peroxide (H_2O_2, 100 μM) was added together. For comparison, 100 μL fresh DMEM^{++} medium containing H_2O_2 (100 μM) was used as a control. After 12 h of cell culture, each group was (or was not) exposed to NIR-II laser (1064 nm) irradiation for 5 min at a power density of 1.0 W/cm^2. After re-incubation for another 4 h, CCK-8 solution (10 μL) was added to each well and cells were incubated for additional 2 h under regular culture conditions. Finally, the absorbance of each well was quantified to evaluate the effect of therapy of TM-P@SPN or TM@SPN, with or without NIR-II laser irradiation.

Cell apoptosis assay and live/dead cell staining assay

The apoptosis of GL261 cells induced by TM-P@SPN and TM@SPN was examined using an Annexin V-FITC/PI apoptosis detection kit. In brief, GL261 cells were seeded in a 6-well plate at a density of 1.5×10^5 cells per well in 1 mL of DMEM^{++} medium overnight. Then, the cell medium was replaced with medium containing TM-P@SPN or TM@SPN ([SP] = 25 μg/mL). Meanwhile, H_2O_2 (100 μM) was added together. The cells treated with 1 mL DMEM^{++} medium containing H_2O_2 (100 μM) were used as control. After 12 h, each group was (or was not) exposed to NIR-II laser (1064 nm) irradiation for 5 min at a power density of 1.0 W/cm^2. After re-incubation for another 4 h, the cells in each well were washed, collected, resuspended

in 195 μL of binding buffer, and added with 5 μL of Annexin V-FITC and 5 μL of PI according to the kit instruction. Then, each sample was incubated for 15 min in the dark before flow cytometry analysis. Cells treated with PBS were used as control. For each sample, 2×10^4 cells were counted, and each measurement was repeated for 3 times. The live/dead cell staining assay was performed using an analogous experimental procedure to the apoptosis assay described above, with the only modification being the use of Calcein-AM/PI instead of Annexin V-FITC/PI for staining.

Determination of GSH contents *in vitro*

GL261 cells were seeded in a 6-well plate at a density of 1.0×10^6 cells per well in 2 mL of DMEM^{++} medium overnight. Then, the medium of each well was replaced with 1 mL fresh DMEM^{++} medium containing TM-P@SPN or TM@SPN ([SP] = 25 μg/mL). Meanwhile, H_2O_2 (100 μM) was added together. And the cells treated with 1 mL DMEM^{++} medium containing H_2O_2 (100 μM) was used as control. After 12 h, each group was (or was not) exposed to NIR-II laser (1064 nm) irradiation for 5 min at a power density of 1.0 W/cm^2. After re-incubation for another 4 h, the cells were washed with PBS for three times. According to supplier's instructions, the cells were examined with commercial GSH assay kit.

Determination of ROS levels *in vitro*

For ROS quantification, GL261 cells were seeded in confocal dishes at a density of 1.0×10^5 cells per well in 0.5 mL of DMEM^{++} medium overnight. Then, the cell medium was replaced with medium containing TM-P@SPN or TM@SPN ([SP] = 25 μg/mL). Meanwhile, H_2O_2 (100 μM) was added together. The cells treated with 0.5 mL DMEM^{++} medium containing H_2O_2 (100 μM) were used as control. After 12 h, each group was (or was not) exposed to NIR-II laser (1064 nm) irradiation for 5 min at a power density of 1.0 W/cm^2. After re-incubation for another 4 h, the cells were washed with PBS for three times before the addition of 1 mL of

serum-free medium containing 10 μM DCFH-DA. After incubation for 40 min at 37 °C in the dark, the cells were washed twice with PBS and analyzed with CLSM.

***In vitro* ICD investigation**

To verify the ICD effect of cancer cells induced by different complexes, the expression of CRT and the release of ATP and HMGB-1 were first detected. In brief, GL261 cells were seeded in 12-well plates at a density of 1×10^5 cells per well with 1 mL of DMEM^{++} and incubated overnight. Then, the culture medium in each well was replaced by fresh DMEM^{++} containing TM-P@SPN or TM@SPN ([SP] = 25 μg/mL). Meanwhile, H_2O_2 (100 μM) was added together. For comparison, 100 μL fresh DMEM^{++} medium containing H_2O_2 (100 μM) was used as a control. After 12 h, each group was (or was not) exposed to NIR-II laser (1064 nm) irradiation for 5 min at a power density of 1.0 W/cm^2. After re-incubation for another 4 h, the cells were washed with PBS for three times, and subjected to CRT immunofluorescence staining followed the standard procedures. While, the culture medium from each well was collected and used for the detection of ATP using an ATP detection kit or HMGB-1 using an HMGB-1 ELISA assay kit according to the manufacturer's instruction, respectively.

Determination of IFN-β levels *in vitro*

DCs were seeded in 12-well plates at a density of 1×10^5 cells per well with 1 mL of DMEM^{++} and incubated overnight. Then, the culture medium in each well was replaced by fresh DMEM^{++} containing TM-P@SPN or TM@SPN ([SP] = 25 μg/mL). Meanwhile, H_2O_2 (100 μM) was added together. After 12 h, each group was exposed to NIR-II laser (1064 nm) irradiation for 5 min at a power density of 1.0 W/cm^2. After re-incubation for another 4 h, the culture medium from each well was collected and used for the detection of IFN-β using an IFN-β ELISA assay kit according to the manufacturer's instruction.

Establishment of an orthotopic glioma model

All animal procedures were conducted in compliance with the ethical guidelines approved by the Animal Care and Use Committee of Donghua University (Approve number: DHUEC-NSFC-2022-16). The female C57BL/6 mice were provided by Shanghai JieSiJie Laboratory Animal Co., Ltd. (China). One week later, mice (≈ 20 g per mouse) were utilized to establish the orthotopic GL261 tumor model. Briefly, the mice were anesthetized by intraperitoneal injection of tribromoethanol (400 μL for each mouse), and then their heads were shaved and aseptically treated with 75% ethanol. After that, the mice were fixed in a stereotactic instrument to have a skin incision over the bregma along the midline. A 27G needle was used to make a burr hole located 1.0 mm front and 2.0 mm left from the bregma. Then, 1×10^6 GL261 cells expressing luciferase (GL261-Luc, in 5 μL PBS) were injected slowly into the brain at a depth of 3.0 mm by a micro syringe. The injection process lasted for 5 min. Finally, the bone wax was used to seal the burr hole, and the skin incision was closed with surgical clips. It is worth mentioning that more than 95% of mice could recover from the surgery, and the incidence of glioma establishment was almost 100%.

***In vivo* FLI and biodistribution**

Mice with orthotopic GL261 tumors were fluorescence scanned. The tumor-bearing mice were intravenously injected with a PBS solution containing the TM-P@SPN or TM@SPN ([SP] = 300 μg/mL, 200 μL for each mouse) before fluorescence imaging. At different time points post-injection (0, 4, 8, 12,16, 24, 36, and 48 h, respectively), the fluorescence intensity of the brains in mice were observed and quantified using a IVIS Spectrum imaging system (PerkinElmer, USA, ex/em = 420/670 nm). In addition, at the time point (12 h) when the fluorescence intensity in the brain was the highest, mice were sacrificed to extract their main organs for FLI *ex vivo*.

MRI

Firstly, TM@SPN and TM-P@SPN solutions were prepared at Mn concentrations of 0.1, 0.3, 0.5, 1.0, and 2.0 mM. And then, these solutions were respectively incubated with GSH (10 mM) for 20 minutes. Subsequently, T_1 MR relaxometry was performed using a 0.5-T NMI20-Analyst NMR analyzing and imaging system (Shanghai Niumag Corporation, Shanghai, China). The parameters were set as follows: IR sequence, resolution = 156 mm × 156 mm, section thickness = 0.8 mm, and excitation number of 1. The r_1 relaxivity was calculated by linearly fitting the inverse T_1 relaxation time ($1/T_1$) as a function of Mn concentration in the end.

Mice with orthotopic GL261 tumors were also MR scanned. The tumor-bearing mice were intravenously injected with a PBS solution containing the TM-P@SPN or TM@SPN ([SP] = 300 μg/mL, 200 μL for each mouse) before MR imaging. MR imaging was performed at different time points post-injection (0, 4, 8, 12, and 24 h, respectively) by a 3.0-T MR system (Siemens Medical Systems, Erlangen, Germany) with an additional custom-built rodent receiver coil (Chenguang Med Tech, Shanghai, China). The instrumental parameters of T_1-weighted MR imaging were set at a spin sequence (TR/TE = 500/15 ms, TE = 10.7 ms, spatial resolution: 0.1 × 0.1 × 0.5 mm^3. Correspondingly, the relative grayscale of tumor sites was quantified using a 3.0-T MR system.

PAI

Frist of all, TM@SPN and TM-P@SPN solutions were prepared at SP concentrations of 12.5, 25, 50, 100, and 200 μg/mL. These solutions were respectively incubated with GSH (10 mM) for 20 min. Subsequently, PA signal intensity was quantified using a 3D optoacoustic imaging system (LOIS-3D, TomoWave Suzhou Medical Imaging Co., Ltd., China) with 1064 nm wavelength. The relative intensity was quantified based on SP concentrations in the end.

For *in vivo* studies, the tumor-bearing mice were intravenously injected with a PBS solution containing the TM-P@SPN or TM@SPN ([SP] = 300 μg/mL, 200 μL for each mouse). PAI was performed at different time points post-injection (0 and 12 h, respectively) by the 3D

optoacoustic imaging system. Correspondingly, the relative intensity of tumor sites was also quantified.

Antitumor therapeutic efficacy in an orthotopic glioma model

For therapy of glioma *in vivo*, after 7 days of successful orthotopic GL261 tumor model preparation, the mice were randomly divided into six groups (n = 5 in each group): PBS, TM@SPN, TM-P@SPN, PBS + laser, TM@SPN + laser, and TM-P@SPN + laser. In each group, PBS, TM@SPN or TM-P@SPN solution ([SP] = 300 μg/mL, 200 μL for each mouse) were injected intravenously into each mouse every 2 days for 3 consecutive times, respectively. And laser irradiation (1064 nm, 1.0 W/cm^2, 5 min) of the brain was also performed every 2 days for 3 consecutive times. Of note, the laser irradiation always occurred on the day after the injection. Tumor progression was monitored *via* bioluminescence imaging (BLI) using an IVIS Spectrum system every 7 days. The body weight and survival status of mice were measured every 2 days.

The tumor after 20 days' treatment in different groups was sectioned into slices for hematoxylin-eosin (H&E) staining. The tumor after the first intravenous injection of different nanoparticles and laser irradiation in each group was sectioned into slices for DCFH-DA fluorescence imaging. On the 7th day after the first intravenous injection of different nanochangers and laser irradiation in each group, the tumor sections were subjected to immunofluorescence staining of HMBG1 and CRT. On the 2th day after the first intravenous injection of different nanoparticles and laser irradiation in each group, the tumor was subjected to test total GSH, ATP, and IFN-β levels, respectively. All operations were carried out in accordance with the manufacturer's instructions.

Distribution of immune cells in TME and spleen

To explore the mechanism of immunotherapy, on the 7th day after the first intravenous injection of different nanoparticles and laser irradiation, tumor-bearing mice in different groups

were sacrificed to extract inguinal lymph nodes, tumor-bearing brain, and spleens. Next, to check the distribution of matured DCs in the lymph nodes, the lymph nodes were cut into small pieces, grinded, and filtered through a 200-mesh sieve to acquire single cell suspension. To examine the distribution of T cells in the spleen of distal tumor model, the spleen tissue was cut into small pieces, grinded, suspended in DMEM, and filtered through a 200-mesh sieve. The filtrated suspension was centrifuged (1000 rpm for 5 min) and resuspended in DMEM. The suspension was filtered by the nylon wool column for 6-8 times to obtain T cells for further analysis. To explore the distribution of T cells in the tumors, the harvested tumor-bearing brain in each group was sectioned into slices.

For the analysis of DCs, the single cell suspension derived from lymph nodes was stained with CD11c-FITC, anti-CD86-APC and anti-CD80-PE antibodies. For the analysis of spleen-infiltrated T cells, the cell suspension was stained with CD45-BV605, anti-CD3- APC/Cy7, anti-CD4-FITC, and anti-CD8β-PE antibodies. For the analysis of spleen-infiltrated regulatory T cells (Tregs), the cell suspension was stained with anti-CD4-FITC, anti-CD25-APC and anti-Foxp3-PE antibodies. Whereafter, Flow cytometry was performed on a BD FACSymphony™ A5 (Franklin Lakes, NJ) with 405/488/561/640 nm laser configuration. And data analysis was conducted using FlowJo v10.7.2 software through boolean gating strategy (SSC-A/FSC-A exclusion). For the analysis of tumor-infiltrated T cells, the tumor tissues were sectioned for immunofluorescence staining of CD4, CD8 and Foxp3.

***In vivo* biosafety investigation**

The histological changes of major organs were also observed using H&E staining. In brief, the mice in different treatment groups were sacrificed at 20 days post-injection, and the vital organs (heart, liver, spleen, lung, and kidney) were excised, fixed in 4% paraformaldehyde overnight, embedded in paraffin, and sectioned for H&E staining. In addition, the mice were randomly divided into six groups: PBS, TM@SPN, TM-P@SPN, PBS + laser, TM@SPN +

laser, and TM-P@SPN + laser. After intravenous injection and laser irradiation were administered to the mice according to the experimental groups, blood samples were collected *via* orbital bleeding and stabilized with heparin for blood routine and blood biochemical analysis, respectively.

References

[1] Wang, M; Xue, F; An, L; Wu, D; Sha, S; Huang, G; et al. Photoacoustic-Imaging-Guided Photothermal Regulation of Calcium Influx for Enhanced Immunogenic Cell Death. Adv Funct Mater. 2024; 34: 2311853.

[2] Qiao, C; Yang, J; Shen, Q; Liu, R; Li, Y; Shi, Y; et al. Traceable Nanoparticles with Dual Targeting and ROS Response for RNAi-Based Immunochemotherapy of Intracranial Glioblastoma Treatment. Adv Mater. 2018; 30: 1705054.
